# From Traditional Efficacy to Drug Design: A Review of Astragali Radix

**DOI:** 10.3390/ph18030413

**Published:** 2025-03-14

**Authors:** Xiaojie Jin, Huijuan Zhang, Xiaorong Xie, Min Zhang, Ruifeng Wang, Hao Liu, Xinyu Wang, Jiao Wang, Dangui Li, Yaling Li, Weiwei Xue, Jintian Li, Jianxin He, Yongqi Liu, Juan Yao

**Affiliations:** 1 College of Pharmacy, Gansu University of Chinese Medicine, Lanzhou 730000, China; jinlovedream@163.com (X.J.); zhj3058743941@163.com (H.Z.); 17339857107@163.com (X.X.); zmandwww@163.com (M.Z.); wxy2181973745@163.com (X.W.); wgg2631106143@163.com (J.W.);; 2 Provincial Key Laboratory of Molecular Medicine and Prevention Research of Major Diseases, Gansu University of Chinese Medicine, Lanzhou 730000, China; wangruifeng0526@163.com (R.W.); liyaling_09@sina.com (Y.L.); hejx4663@gszy.edu.cn (J.H.); 3 Key Laboratory of Dunhuang Medicine, Ministry of Education, Gansu University of Traditional Chinese Medicine, Lanzhou 730000, China; ljt@gszy.edu.cn; 4 School of Basic Medicine, Gansu University of Traditional Chinese Medicine, Lanzhou 730000, China; 5 Innovative Drug Research Centre, School of Pharmaceutical Sciences, Chongqing University, Chongqing 404100, China; xueww@cqu.edu.cn

**Keywords:** Astragali Radix, review, botanical characteristics, phytochemistry, pharmacology, drug design

## Abstract

Astragali Radix (AR), a traditional Chinese herbal medicine, is derived from the dried roots of *Astragalus membranaceus* (Fisch.) Bge. var. *mongholicus* (Bge.) Hsiao (*A. membranaceus* var. mongholicus, AMM) or *Astragalus membranaceus* (Fisch.) Bge (A. *membranaceus*, AM). According to traditional Chinese medicine (TCM) theory, AR is believed to tonify qi, elevate yang, consolidate the body’s surface to reduce sweating, promote diuresis and reduce swelling, generate body fluids, and nourish the blood. It has been widely used to treat general weakness and chronic illnesses and to improve overall vitality. Extensive research has identified various medicinal properties of AR, including anti-tumor, antioxidant, cardiovascular-protective, immunomodulatory, anti-inflammatory, anti-diabetic, and neuroprotective effects. With advancements in technology, methods such as computer-aided drug design (CADD) and artificial intelligence (AI) are increasingly being applied to the development of TCM. This review summarizes the progress of research on AR over the past decades, providing a comprehensive overview of its traditional efficacy, botanical characteristics, drug design and distribution, chemical constituents, and phytochemistry. This review aims to enhance researchers’ understanding of AR and its pharmaceutical potential, thereby facilitating further development and utilization.

## 1. Introduction

Astragalus Radix (AR), a member of the Astragalus genus within the Fabaceae family, holds a prestigious status in TCM. Classified as a “top-grade” herb in the ancient *Shen Nong’s Materia Medica*, AR has been valued for its medicinal properties for centuries. According to the 2020 edition of the Pharmacopoeia of the People’s Republic of China, AR refers to the dried roots of AM [1]. It has a sweet flavor and warm nature, with meridian tropism for the lungs and spleen. In TCM theory, AR is believed to tonify qi, raise yang, consolidate the exterior to stop sweating, promote diuresis and reduce swelling, generate body fluids, nourish the blood, alleviate stagnation, relieve arthralgia, expel toxins and pus, and facilitate wound healing. Due to these therapeutic effects, AR is widely used in TCM formulations to treat general weakness and chronic illnesses and enhance overall vitality [2]. Modern pharmacological studies have further validated AR’s diverse bioactive properties. Research has demonstrated its anti-tumor, antioxidant, cardiovascular-protective, immunomodulatory, anti-inflammatory, anti-diabetic, and neuroprotective effects [3,4,5,6,7,8,9]. Additionally, AR has gained attention in the food industry, with its incorporation into products such as biscuits and beverages, thereby expanding its applications beyond traditional medicine [10].

From virtual screening to drug design, computational and artificial intelligence-based methods are increasingly utilized in TCM research. The identification of lead compounds from extensive compound libraries is a critical aspect of TCM research and development [11]. Currently, TCM research methods focus on reverse prediction, protein-protein interaction networks, molecular docking, virtual screening, target fishing, machine learning, and other computational approaches [12,13,14]. These methods are closely associated with the chemical constituents of TCM. The constituent structure of TCM is multi-source and available in online databases [15]. The structural composition of TCM constituents is diverse and supported by online databases [16]. This study provides a more comprehensive structural analysis of AR constituents, facilitating further research on AR and the identification of lead compounds. These findings contribute to the development of novel drugs and the advancement of intelligent drug design for AR, ultimately promoting the integration of traditional and modern medicine and accelerating drug discovery [17].

In this comprehensive review, we aim to provide an updated and detailed overview of AR, covering its traditional efficacy, botanical characteristics, distribution, chemical constituents, phytochemistry, and role in drug design. Given that existing reviews on AR are often fragmented or lack sufficient detail, we seek to present a more thorough and systematic summary of recent advancements in AR research. Specifically, we examine its botanical characteristics, distribution, chemical constituents, phytochemistry, and potential applications in drug design. Furthermore, by systematically reviewing its chemical constituents, this study aims to guide the discovery of lead compounds and the development of novel drugs.

## 2. Methods

It is crucial to delve deeper into the various studies and advancements made in recent years to further understand the research progress of AR. Data were obtained from resources including CNKI, PubMed, and ScienceDirect. Keyword searches for information included “Astragali Radix”, “*Astragalus membranaceus* (Fisch.) Bge. var. *mongholicus* (Bge.) Hsiao”, “*Astragalus membranaceus* (Fisch.) Bge”, “pharmacologic actions”, “chemical constituents”, “traditional efficacy”, “botany”, “preparation”, “virtual filtering”, “lead compound”. At the same time, the related research of each part was deeply investigated. This review encompasses 332 references spanning September 1983 to 2024.

## 3. Traditional Efficacy

AR is one of the most renowned herbal medicines and is recognized as the foremost qi-boosting tonic in China. In TCM theory, qi is considered a fundamental substance that constitutes the human body and sustains essential life activities [18]. Due to its extensive clinical applications and well-documented therapeutic effects, AR continues to be widely used. AM is primarily prescribed for qi deficiency and fatigue, whereas AMM is used to address qi and blood deficiencies. AR is often combined with various herbs, such as *Angelica sinensis* and *Glycyrrhiza uralensis*, to enhance its medicinal efficacy [19]. For instance, Danggui Buxue Decoction, a formulation consisting of AR and Angelica sinensis, is traditionally used to tonify qi and blood in individuals with blood deficiency. The ratio of AR to *Angelica sinensis* in this formula is 5:1, reflecting the TCM principle that qi generates blood. A detailed description of this decoction can be found in the ancient medical text, Lan Shi Mi Zang. Modern research has demonstrated that Danggui Buxue Decoction plays a role in immune regulation [20,21], and AR has a long history of application. It is widely used in clinical practice. The relevant classic formulations and listed drugs are presented in Table 1.

TCM processing, which has a long history, represents the essence of TCM applications [22]. According to the different processed methods of AR, it can be categorized into raw AR and processed AR. Raw AR refers to the dried slices of AR root, which are effective in fixing the surface and stopping sweating, supporting sores and generating muscle, promoting diuresis, and detumescence. It is commonly used for general health maintenance and treatment. Honey-processed Astragalus Radix is a representative product of processed AR, typically denoting a processing method in which AR is stir-fried with refined honey. It is prepared by stir-frying sliced AR with honey [23]. Honey-processed Astragalus Radix excels at tonifying qi and generating blood, mainly tonifying middle qi, and is primarily used to treat qi deficiency and fatigue, less food, and loose stool [23,24]. Depending on the specific conditions of different patients, various processed AR products can be selected to optimize treatment effectiveness.

Due to its exceptional tonic properties, AR is widely regarded as both a medicinal and dietary ingredient, highlighting its dual role in healthcare and nutrition. However, wild AR resources are becoming increasingly scarce due to excessive harvesting and environmental degradation. With the rising demand for AR, which has recently exceeded supply, artificially cultivated varieties such as AM and AMM have emerged as the primary medicinal sources. AR is frequently incorporated into daily diets as a condiment and can also be consumed as a tea substitute. Moreover, when combined with *Jujubae Fructus*, it forms Astragalus tea, which is believed to strengthen the spleen and enhance immunity. These edible applications underscore the significant potential for the further development and utilization of AR in functional foods.

**Table 1:** Classic formulations and marketed drugs for AR.

Classic Formulations	Main Composition	Traditional and Clinical Uses	Marketed Drugs	References
Buzhong Yiqi Decoction	Astragali Radix, Ginseng Radix.et Rhizoma, Cimicifugae Rhizoma	spleen asthenia, prolapse of anus, sagging of viscera	Buzhong Yiqi Wan, Buzhong Yiqi Mixture, Buzhong Yiqi granule	[1,25]
Yupingfeng san	Astragali Radix, Atractylodis Macrocephalae Rhizoma, Saposhnikoviae Radix	Superficial asthenia, spontaneous sweating	Yupingfeng Wan, Yupingfeng Granule, Yupingfeng oral liquid	[1,25]
Danggui Buxue Decoction	Astragali Radix, Angelicae Sinensis Radix	blood-deficiency fever	Dangguibuxue Wan, Danggui buxue capsule, Danggui buxue oral liquid	[1,25]
Guipi Decoction	Astragali Radix, Ginseng Radix.et Rhizoma, Atractylodis, Macrocephalae Rhizoma	qi and blood deficiency, morbid forgetfulness insomnia, night-sweat	Guipi Wan, Guipi Ointment	[1,25]
Huangqi Jianzhong Decoction	Astragali Radix, Cerealose, Cinnamomi Ramulus	deficiency of vital energy internal cold, Abdominal urgent pain	Huangqi Jianzhong Wan	[1,25]
Baoyuan Decoction	Astragali Radix, Ginseng Radix.et Rhizoma, Cinnamomi Cortex	Variola, Qi deficiency subsidence	Baoyuan Wan	[1,25]
Bufei Decoction	Astragali Radix, Asteris Radix et Rhizoma, Ginseng Radix.et Rhizoma	pulmonary asthenia, cough with asthma, short breath, spontaneous sweating	Bufei Wan	[1,25]
Yuye Decoction	Astragali Radix, Trichosanthis Radix, Puetaaiae Lobatae Radix	thirst	Yuye wan, Yuye Xiaoke Granules, Yuye Xiaoke Granules	[1,25]
Buyang Huanwu Decoction	Astragali Radix, Angelicae Sinensis Radix, Chuanxiong Rhizoma	blood stasis, half-length-flabbiness		[1,25]
Shiquandabu Decoction	Astragali Radix, Ginseng Radix.et Rhizoma, Angelicae Sinensis Radix, Cinnamomi Cortex	sallow complexion, Knee weakness, deficiency of qi and blood, Various kinds of weakness	Shiquan Dabu Ointment Shiquan Dabu Wan Shiquan Dabu tabella sze chuan dah boochiew	[1,25]
Renshen yangrong wan	Astragali Radix, Cinnamomi Cortex, Ginseng Radix.et Rhizoma, Atractylodis Macrocephalae Rhizoma	deficiency of heart and spleen, deficiency of qi and blood, poor appetite and loose stools, Weakness after illness	Renshen Yangrong Wan	[1,25]
Fangji Huangqi Decoction	Astragali Radix, Stephaniae Tetrandorae Radix, Glycyrrhizae Radix et Rhizoma	wind damp syndrome, Limb pain, difficult urination	—	[1,25]
Huangqi guizhi wuwu Decoction	Astragali Radix, Paeoniae Radix Alba, Cinnamomi Ramulus	blood arthralgia, flesh benumbed and unresponsive	—	[1,25]
Tuolitounong san	Astragali Radix, Ginseng Radix.et Rhizoma, Anglicae Dahuricae Radix	superficial infection invade into cerebral carbuncle pus hard to rupture	—	[1,25]

## 4. Botanical Characteristics and Distribution

**Figure 1:**
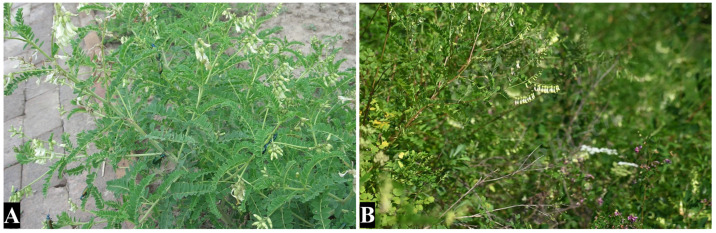
The botanical of AR including AMM (**A**), AM (**B**), URL: http://ppbc.iplant.cn/ (accessed on 1 March 2025), photo A ID: 1649885, photo B ID: 15508236.

AM and AMM are the original species and variations of the same species (Figure 1). Both are considered authentic AR in the 2020 edition of the *Chinese Pharmacopoeia*, and they have close genetic relationships. Some differences have been observed between them in terms of their botanical features. Both species are perennial herbs with stout taproots, woody, upright stems, and pinnately compound leaves. The flower is raceme, and the bracts are linear and lanceolate. The pedicel is about 3–4 mm in length and is densely covered with black pubescence. The calyx is bell-shaped, and the corolla is pale yellow and about 12–20 mm in length, forming a papilionaceous. There are 10 stamens, forming diadelphous stamens. Pods are membranous and ovate-oblong, and the seeds are 5–6, kidney-shaped, and black. The flowering period is from June to July, and the fruiting period is from August to September [19]. Although the two plants have great similarities in botanical characteristics, there are few differences in height of stems, the shapes and size of leaves, the number of seeds and other aspects. The root is the main medicinal part of AR. The taste is slightly sweet. The root is cylindrical, about 40–100 cm long, with few branches, some of which are slightly distorted. The root head is slightly enlarged, the surface is grayish yellow or light brown, the cortex is yellowish white, and the xylem is light yellow, with a radial texture and or fissures. There is a beany smell when chewing. Due to the different colors of the roots, AMM is called “Heipiqi” in the commodity, and AM is called “Baipiqi” [26] (Table 2).

AR is mainly produced in Inner Mongolia, Shanxi, Gansu, Heilongjiang, and other places in China, and also grows in Sichuan, Yunnan, Jilin, Hebei, and other places. Due to the influence of climate, temperature, and other environmental factors, the chemical constituents of AR in different regions are not the same [27]. The genuine AR used in clinics is the dry root of AMM or AM. Due to the profit in the sale process, there are many adulterants in AR. After processing, the adulterants are more similar to genuine AR in appearance and shape, and it is difficult to distinguish the true and false, which undoubtedly causes great hidden dangers to the drug efficacy and drug safety of consumers. At present, the common adulterants of AR in the market are mainly *Hedysari Radix*, *Medicago sativa* L., A *stragalus ernestii* Comb., *Malva rotundifolia* L., *Gossypium herbaceum* L., etc. The common confusions of AR are listed in Table 3. Therefore, clinical medication needs to do a good job of quality control [28].

**Table 2:** The difference between AMM and AM.

Species	Leaf	Corolla	Ovary	Fruit	Root	References
*Astragalus membranaceus* (Fisch.) Bge. var. ***mongholicus*** (Bge.) Hsiao	25–37 leaflets, and broadly elliptical small leaves; 5–9 mm long, width 3–5 mm, with short white pubescence	light yellow	glabrous	Ovate oblong; no pubescence	30–90 cm long, surface brown bitumen	[19,26]
*Astragalus membranaceus* (Fisch.) Bge.	13–31 leaflets, and oval or oblong ovate, small leaves; 4–10 mm long, width 3–5 mm, with short white pubescence	yellow	puberulous	half the oval; having white or black pubescence	40–80 cm long, surface brown taupe	[19,26]

**Table 3:** The common adulterants of AR.

Variety	Taxonomic Position	Root Traits	References
*Medicago sativa* L.	Leguminosae, *Medicago* L.	Bitter flavor, pungent smell, cylindrical root, crisp texture, easy to break. The section is strong in fiber and the forming ring is not obvious.	[28]
*Astragalus ernestii* Comb.	Leguminosae, *Astragalus* L.	Weak taste, the root is thick, the texture is loose and flexible. The section has strong fiber and the skin is easy to fall off.	[28,29]
*Oxytropis coerulea* (Pall.) DC.	Leguminosae, *Oxytropis* DC	Weak taste, cylindrical root with branches, surface brown yellow or brown red, light and tough, strong toughness.	[30,31]
*Caragana sinica* (Buchoz.) Rehd.	Leguminosae, *Caragana* Fabr.	Weak taste, cylindrical root, surface brown yellow, brittle and easy to break.	[30,32]
*Melilotus albus* Desr.	Leguminosae, *Melilotus* (L.) Mill.	Weak taste, cylindrical root, with a swollen head, surface brown yellow brown to red brown surface. The root is hard and brittle, and the section is prickly.	[30]
*Glycyrrhiza pallidiflora* Maxim.	Leguminosae, *Glycyrrhiza* L.	Sweet flavor, cylindrical root, the head has more branches, surface brown grayish yellow to grayish brown. The texture is hard to break, and the section is fibrous.	[33,34]
*V.gigantea* Bge.	Leguminosae, *Pisum* Linn	Bitter flavor, cylindrical root, hard and crisp, surface brown yellowish white, yellowish yellow in wood.	[35,36]
*Malva rotundifolia* L.	Malvaceae, *Malva* Linn.	Sweet flavor, cylindrical root, cylindrical root, multi-branched, surface brown earthy yellow, hard and brittle, easy to break. The section is fibrous and flat.	[32]
*Althaea rosea* (L.) Cavan	Malvaceae, *Althaea* Linn	Sweet flavor, cylindrical root, the root head is coarse. The lower end is fine, surface brown yellowish brown. The texture is hard, and the section is not neat.	[33,37]
*Malva verticillata* L.	Malva *verlicillala* L.	Sweet flavor, cylindrical root, surface brown light yellowish, with longitudinal stripes. The section is yellowish white.	[28]
*Hedysarum polybotrys* Hand.-Mazz.	Malvaceae, *Malva* Linn.	Sweet flavor, cylindrical root, surface brown gray reddish brown, and the texture is hard and tough, sectional fiber.	[28]
*Astragalus tongolensis* Ulbr.	Leguminosae, *Astragalus* L.	Sweet flavor, surface brown epidermis yellowish to brownish brown. The texture is loose and flexible, not easy to break, sectional fiber is weak.	[28]
*Astragalus floridulus* Podlech	Leguminosae, *Astragalus L.*	Sweet flavor, epidermis dark brown, hard and tough texture. Section fibrous, weak powder, narrow skin.	[32]
*Astragalus chrysopterus* Bunge	Leguminosae, *Astragalus* L.	Sweet flavor, rhizome is thick, surface brown yellowish brown. The texture is dense and tough. Section fiber, powder-rich.	[28]
*Sphaerophysa salsula* (Pall.) DC.	Leguminosae, *Sphaerophysa* DC	Bitter flavor, the root is multi-branched, with small poison and semi-shrub. crisp and easy to break. The section is neat. poor fiber, no bean smell.	[38]
*Gossypium hirsutum* Linn.	Malvaceae, *Gossypium* Linn.	Bitter flavor, the roots are cylindrical, with small branches at the lower part, surface brown yellow or light brown. Hard and light, not easy to break, Sectional fiber.	[26]

## 5. Chemical Constituents of AMM and AM

AR is a prominent herbal medicine noted for its diverse constituents and multi-faceted activities, with a wide geographical distribution. Among the various types of Astragalus, AMM and AM are the two most commonly used medicinal applications. Although the primary concentration of chemical constituents is derived from the root, some active constituents can also be extracted from the flowers of AR, which possess certain anti-oxidation effects [39]. To date, researchers have isolated over 300 constituents from AMM and AM, comprising 184 flavonoids, 101 saponins, and 42 other constituents.

This study aims to furnish a more comprehensive understanding of the chemical constituents found in AR through an extensive literature search [40,41,42]. Detailed information is presented in the tables below.

### 5.1. Flavonoids

**Figure 2:**
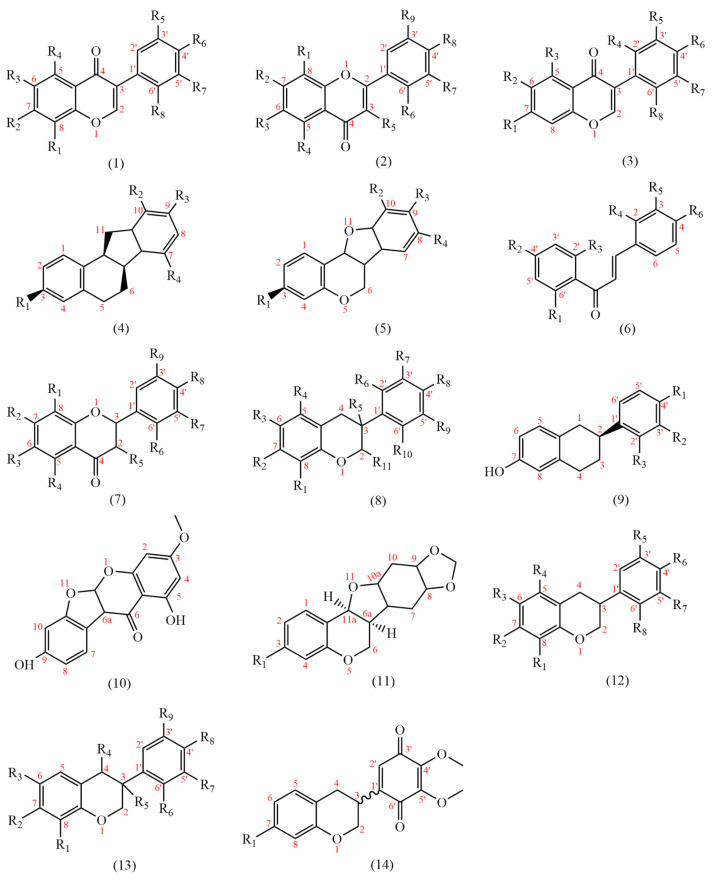
The structural backbones of flavonoids in AM and AMM.

At present, 183 flavonoids have been isolated from AMM and AM (Table 4); AMM contained 153 flavonoids, and AM contained 69 flavonoids, including 38 common flavonoids. The diversity of flavonoids in AMM significantly surpasses that of AM. The structures of the flavonoid skeletons from AM and AMM are shown in Figure 2.

**Figure 3:**
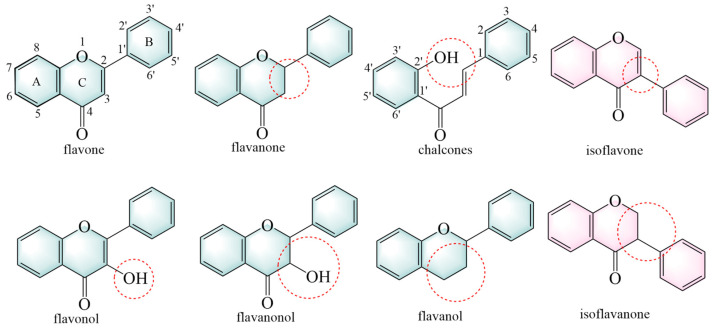
The structural backbones of flavonoids. The red circle indicates the site of modification in the flavonoid’s parent nucleus.

Flavonoids are primarily classified based on their chemical structures into several major subclasses, including flavones, flavanones, chalcones, isoflavones, flavanols, flavanonols, and isoflavanone (Figure 3) [40].

AR contains many flavones (**54**–**61**) based on 2-phenylchromone as the basic structural backbone. Flavones have anti-diabetic [43], neuroprotective [44], and anti-inflammatory properties [45]. Apigenin (**58**) is a typical flavonoid component that exerts protective effects on the liver [46] and vasculature [47]. It also has a certain curative effect on Alzheimer’s disease [48] and acute lymphoblastic [49].

Flavonols (**62**–**95**) represent a class of constituents characterized by the presence of a hydroxyl group at the 3-position of the 2-phenylchromone backbone. It can treat cardiovascular diseases, with anti-tumor [50], anti-oxidation [51], and other effects [50].

AR contains many isoflavones (**1**–**53**, **96**–**100**), and 3-phenylchromone is the basic structural backbone of isoflavones. It can be antibacterial [52] and anti-inflammatory [53]. Genistein (**4**) can anti-cancer [54], anti-neurodegenerative diseases [55], and relieve rheumatoid osteoarthritis [56]. Formononetin (**1**) is a derivative of isoflavones. It has anti-inflammatory [57] and neuroprotective effects [58] and can ameliorate polycystic ovary syndrome [53].

Isoflavanones are products resulting from the hydrogenation reduction of the 2,3 double bonds in 3-phenylchromone. Isoflavanones (**107**–**126**, **164**, **166**) have anti-tumor effects [59].

Chalcones are constituents formed by the cleavage of the chemical bond between positions 1 and 2 of 2-phenylchromone, resulting in a benzaldehyde-condensing acetophenone structure, and their 2′-hydroxy derivative is an isomer of isoflavanone [40]. Chalcones (**127**–**133**) can be converted into dihydroflavones under acidic conditions. Chalcones have anti-oxidatant, antibacterial [60], neuroinflammation-relieving [61], and anti-cancer effects [62].

Flavanones (**134**–**138**) are formed by the hydrogenation of the 2, 3 double bonds in 2-phenylchromone. Naringin (**135**) is a common flavanone, which can protect blood vessels [63] and has anti-atherosclerotic properties [64].

**Table 4:** Flavonoids isolated from AM and AMM.

No.	Name	Substituent	Skeletons	Species	References
**1**	formononetin	R_2_=OH R_6_=OMe	1	AMM, AM	[65,66]
**2**	calycosin	R_2_=OH R_7_=OH R_6_=OMe	1	AMM, AM	[65,66]
**3**	calycosin-7-O-β-D-glucopyranoside	R_2_=β-D-OGlcp R_7_=OH R_6_=OMe	1	AMM, AM	[65,66]
**4**	genistein	R_2_=R4=R_6_=OH	1	AMM, AM	[66,67]
**5**	ononin	R_2_=β-D-OGlcp R_6_=OMe	1	AMM, AM	[66,68]
**6**	6″-acetylononin	R_2_=β-D-(6-acetxyl)-OGlcp R_6_=OMe	1	AMM, AM	[68,69]
**7**	pratensein	R_2_=R_4_=R_7_=OH R_6_=OMe	1	AMM, AM	[70]
**8**	3′-methoxy-5′-hydroxy-isoflavone-7-O-β-D-glucopyranoside	R_1_=β-D-OGlcp R_7_=OMe R_5_=OH	1	AMM, AM	[71]
**9**	odoratin-7-O-β-D-glucopyranoside	R_2_=β-D-OGlcp R_4_=R_6_=OMe R_7_=OH	1	AMM, AM	[69,71]
**10**	daidzein	R_2_=R_6_=OH	1	AMM, AM	[72,73]
**11**	(3R)-2′-hydroxy-7,3′,4′trimethoxy-isoflavan	R_1_=R_4_=R_5_=OMe R_3_=OH	1	AM	[74]
**12**	isomucronulatol	R_2_=OH R_5_=R_6_=OMe R_7_=OH	1	AMM, AM	[68,75]
**13**	isomucronulatol-7,2′-di-O-glucoside	R_2_=R_8_=OGlcp R_5_=R_6_=OMe	1	AMM, AM	[73,75]
**15**	calycosin7-O-(6-O-acety1-β-D-glucopyranoside)	R_2_=β-D-O-(6-O-Ac-Glcp) R_6_=OMe R_7_=OH	1	AMM, AM	[76]
**16**	(3R)-7-O-β-glc-isomucronulatol	R_2_=β-D-OGlcp R_8_=OH R_6_=R_7_=OMe	1	AMM, AM	[76]
**17**	pratensein-7-O-β-D-glycoside	R_2_=β-D-OGlc R_4_=R_7_=OH R_6_=OMe	1	AMM	[66]
**18**	sissotrin	R_2_=β-D-OGlcp R_2_=R_4_=OH R_6_=OMe	1	AMM	[77]
**19**	5′,7-dihydroxy-3′-methoxyisoflavone	R_2_=R_5_=OH R_7_=OMe	1	AMM	[78]
**20**	5,7,4′-trihydroxy-3′-methoxyisoflavone	R_2_=R_4_=R_6_=OH R_7_=OMe	1	AMM	[77]
**21**	4′-methoxyiso-flavone-7-O-β-D-glucopyranoside	R_2_=β-D-OGlcp R_6_=OMe	1	AMM	[79]
**22**	7,3′-diohydroxy-5′-methoxyisoflavone	R_2_=R_5_=OH R_7_=OMe	1	AMM	[79]
**23**	3′-hydroxy-4′-methoxy-7-O-(6″-butylene beaser-O)-β-glucopyranoside	R_2_= [6-(E)-But-2-enoyl]-β-D-OGlcp R_7_=OH R_6_=OMe	1	AMM	[80]
**24**	5′-hydroxy-3′-methoxy-isoflavone-7-O-β-D-glucoside	R_2_=β-D-OGlcp R_7_=OMe R_5_=OH	1	AMM	[78]
**25**	sophorabioside	R_2_=R_4_=OH R_6_=β-D-OGlcp-(2→1)-α-L-Rha	1	AMM	[69]
**26**	odoratin	R_2_=OH R_3_=R_6_=OMe	1	AMM	[81]
**27**	calycosin 7-O-(6-O-malony1-β-D-glucopyranoside)	R_2_=O-(6-O-malonyl-β-D-Glcp) R_7_=OH R_6_=OMe	1	AMM	[76]
**28**	formononetin 7-O-(6-O-malony1-β-D-glucopyranoside)	R_2_=O-(6-O-malonyl-β-D-Glcp) R_7_=OH	1	AMM	[76]
**29**	formononetin 7-O-(6-O-acety1-β-D-glucopyranoside)	R_2_=O-(6-O-Ac-β-D-Glcp) R_7_=OH	1	AMM	[76]
**30**	calycosin 7-O-(6-O-butanoyl-β-D-glucopyranoside)	R_2_=O-(6-O-butanoyl-β-D-Glcp) R_6_=OMe R_7_=OH	1	AMM	[76]
**31**	5′,7-di-OH-3′-methoxyisoflavone	R_2_=R_5_=OH R_7_=OMe	1	AMM	[76]
**32**	calycosin-7-O-glc-6″-O-acetate	R_2_=β-D-OGlcp-Ac R_7_=OH R_6_=OMe	1	AMM	[82]
**33**	dihydroxy-dimethoxy isoflavone	R_3_=R_7_=OMe R_4_=R_8_=OH	1	AMM	[82]
**34**	formononetin-7-O-glc-6″-O-acetate	R_2_=β-D-OGlcp-Ac R_7_=OH R_6_=OMe	1	AMM	[82]
**35**	calycosin-Glc-malonate	R_4_=β-D-OGlcp-Mal R_7_=OH R_6_=OMe	1	AMM	[82]
**36**	calycosin-7-O-Glc-6″-O-malonate	R_2_=β-D-OGlcp-Mal R_7_=OH R_6_=OMe	1	AMM	[82]
**37**	odoratin-7-O-Glc-6″-O-malonate	R_2_=β-D-OGlcp-Mal R_3_=R_6_=OMe R_7_=OH	1	AMM	[82]
**38**	6,4′-Dimethoxyisoflavone-7-O-Glc	R_1_=β-D-OGlcp R_3_=R_6_=OMe	1	AMM	[82]
**39**	pratensein-7-O-Glc-6″-O-malonate	R_2_=β-D-OGlcp-Mal R_4_=R_7_=OH R_6_=OMe	1	AMM	[82]
**40**	formononetin-7-O-Glc-6″-O-malonate	R_2_=β-D-OGlcp-Mal R_6_=OMe	1	AMM	[82]
**41**	7-Hydroxy-6,4-dimethoxyisoflavone	R_3_=OH R_4_=R_6_=OMe	1	AMM	[82]
**42**	3′-Hydroxy-6′,4′-dimethoxyisoflavone-7-O-Glc	R_2_=β-D-OGlcp R_7_=OH R_1_=R_6_=OMe	1	AMM	[82]
**43**	2′,3′-dihydroxy-7,4′-dimethoxyisoflavone	R_2_=R_6_=OMe R_8_=R_7_=OH	1	AMM	[82]
**44**	7,3′-dihydroxy-8,4′-dimethoxyisoflavone	R_2_=R_5_=OH R_1=_R_6_=OMe	1	AM	[65]
**45**	calycosin 7-O-β-D-(6″-acetyl)-glucoside	R_2_=(6″-acetxyl)-β-D-OGlcp R_6_=OMe	1	AM	[68]
**46**	calycosin 7-O-β-D-{6″-[(E)-But-2-enoyl]}-glucoside	R_2_=[6-(E)-But-2-enoyl]-β-D-OGlcp R_6_=OMe	1	AM	[68]
**47**	8,3′-dihydroxy-7,4′-dimethoxyisoflavone	R_2_=R_6_=OMe R_1_=R_7_=OH	1	AM	[65]
**48**	ammopiptanoside A	R_2_=[6-(E)-But-2-enoyl]-OGlcp R_6_=OMe	1	AM	[68]
**49**	3′,7,8-trihydroxy-4′-methoxyisoflavone	R_1_=R_2_=R_7_=OH R_6_=OMe	1	AM	[70]
**50**	glycitein	R_2_=R_8_=OH R_4_=OMe	1	AM	[83]
**51**	4′,7-dihydroxy-3′-methoxy isoflavone	R_2_=R_6_=OH R_7_=OMe	1	AM	[83]
**52**	genistin	R_2_=β-D-OGlcp R_8_=OH	1	AM	[83]
**53**	glycitin	R_2_=β-D-OGlcp R_3_=OMe R_8_=OH	1	AM	[83]
**54**	(6aR,11aR)-3-OH-9,10-dimethoxypterocarpan	R_1_=OH R_2_=R_3_=OMe	2	AMM	[76]
**55**	astrapterocarpan 3-O-(6-O-malony1-β-D-glucopyranoside)	R_1_=O-(6-O-malonyl-β-D-Glcp) R_2_=R_3_=OMe	2	AMM	[76]
**56**	oroxylin A	R_2_=R_4_=OH R_3_=OMe	2	AMM	[84]
**57**	wogonin	R_1_=OMe R_2_=R_4_=OH	2	AMM	[84]
**58**	apigenin	R_2_=R_4_=R_8_=OH	2	AMM	[82]
**59**	baicalein	R_2_=R_3_=R_4_=OH	2	AMM	[82]
**60**	baicalin	R_2_=β-D-OGlcp R_3_=R_4_=OH	2	AMM	[82]
**61**	Oroxylin A	R_2_=R_4_=OH R_3_=OMe	2	AMM	[84]
**62**	kaempferide	R_2_=R_4_=R_5_=OH R_8_=OMe	2	AMM	[82]
**63**	kaempferol	R_2_=R_4_=R_5_=R_8_=R_9_=OH	2	AMM, AM	[41,69]
**64**	rhamnocitrin-3-O-β-D-glucopyranoside	R_2_=R_8_=OH R_4_=OMe R_5_=β-D-OGlcp	2	AMM, AM	[69,83]
**65**	rhamnocitrin-3-O-β-neohesperidoside	R_2_=R_4_=R_8_=OH R_5_=β-D-Rha-(1→2)-OGlcp	2	AMM, AM	[69,83]
**66**	complanatuside	R_2_=OMe R_4_=OH R_8_=R_5_=β-D-OGlcp	2	AMM, AM	[69,83]
**67**	isoquercitrin	R_4_=OH R_2_=OMe R_8_=R_5_=β-D-OGlcp	2	AMM, AM	[41]
**68**	quercetin	R_3_=R_4_=R_5_=R_8_=R_7_=OH	2	AMM, AM	[41,77]
**69**	quercetin-3-glucoside	R_2_=R_4_=R_9_=R_8_=OH R_5_=β-D-OGlcp	2	AMM, AM	[71,77]
**70**	isorhamnetin	R_2_=R_4_=R_5_=R_8_=OH R_7_=OMe	2	AMM, AM	[41,77]
**71**	astraflavonoid B	R_2_=(5′-R)-OApi R_4_=R_8_=OH R_5_=β-D-OGlcp-(2→1)-α-L-Rha	2	AMM	[69]
**72**	kaempferol-3-O-β-D-glucoside	R_2_=R_4_=R_8_=OH R_5_=β-D-OGlcp	2	AMM	[69]
**73**	kaempferol-3,7-di-O-β-D-glucopyranoside	R_2_=R_5_=β-D-OGlcp R_4_=R_8_=OH	2	AMM	[69]
**74**	quercetin-3-O-β-D-neospheroside	R_2_=R_4_=R_7_=R_8_=OH R_5_=β-D-OGlcp-(2→1)-L-Rha	2	AMM	[69]
**75**	tamarixin	R_2_=R_4_=R_7_=OH R_5_=β-D-OGlcp R_8_=OMe	2	AMM	[85]
**76**	isorhamnetin-3-β-D-glucoside	R_2_=R_4_=R_8_=OH R_5_=β-D-OGlcp R_9_=OMe	2	AMM	[81]
**77**	4′-methoxy-kaempferol 3-O-glucoside	R_2_=R_4_=OH R_8_=OMe R_5_=β-D-OGlcp	2	AMM	[81]
**78**	kumatakenin	R_2_=R_5_=OMe R_4_=R_8_=OH	2	AMM	[81]
**79**	Rhamnocitrin	R_4_=R_5_=R_8_=OH R_2_=OMe	2	AMM	[76]
**80**	3-O-β-D-glc-isorhamnetin	R_2_=R_4_=R_8_=OH R_9_=OMe R_5_=β-D-OGlcp	2	AMM	[76]
**81**	5,2′,6′-Trihydroxy-6,7,8-trimethoxyflavone	R_1_=R_2_=R_3_=R_5_=R_6_=OMe R_4_=OH	2	AMM	[82]
**82**	apigenin-Hex	R_2_=R_4_=R_5_=R_7_=OH R_6_=OMe	2	AMM	[82]
**83**	rhamnocitrin-Hex	R_2_=OMe R_4_=R_8_=OH R_5_=β-D-OGlc	2	AMM	[82]
**84**	rhamnocitrin-Hex-malonateHex Hex	R_2_=OMe R_4_=R_8_=OH R_5_=OGlcp	2	AMM	[82]
**85**	rhamnocitrin-Hex-acetate	R_2_=OMe R_4_=R_8_=OH R_5_=β-D-OGlcp-Ac	2	AMM	[82]
**86**	hyperoside	R_2_=R_4_=R_8_=R_7_=OH R_5_=β-D-OGlcp	2	AMM	[82]
**87**	isorhamnetin-3-O-neohespeidoside	R_2_=R_4_=R_7_=OH R_5_=OH-Neohesperidin R_6_=OMe	2	AMM	[82]
**88**	3-hydroxydihydroisoflavone	R_2_=R_4_=R_8_=OH R_5_=OMe	2	AMM	[82]
**89**	Quercetin-3-O-robinobioside	R_2_=R_4_=R_7_=R_8_=OH R_5_=OH-Roeinobioside	2	AMM	[82]
**90**	kaempferol-3-O-rutinoside	R_2_=R_4_=R_8_=OH R_5_=OH-Rutinoside	2	AMM	[82]
**91**	kaempferol-3-O-Glucosyl galactoside	R_5_=OGlcp-Gal R_2_=R_4_=R_8_=OH	2	AMM	[82]
**92**	kaempferol-4′-methoxy-3-O-glucopyranoside	R_2_=R_4_=OH R_8_=OMe R_5_=β-D-OGlcp	2	AMM	[82]
**93**	7-Methoxy-Kaempferol-3-O-Glc	R_2_=OMe R_4_=R_8_=OH R_5_=β-D-OGlcp	2	AMM	[82]
**94**	rhamnocitrin-3-O-β-D-glucopyranoside (1″→2″)-β-D-apiofuranosy	R_4_=R_8_=OH R_2_=OMe R_5_=β-D-OGlcp-(2→1)-Api	2	AM	[83]
**95**	tiliroside	R_4_=R_2_=R_8_=OH R_5_=(6″-p-coumaroyl)-β-D-Glcp	2	AM	[83]
**96**	dihydroxy-trimethoxy DHIF	R_3_=R_8_=OH R_2_=R_7_=R_6_=OMe	3	AMM	[82]
**97**	dihydroxy-trimethoxy DHIF-Hex	R_3_=β-D-OGlcp R_2_=R_7_=R_6_=OMe R_8_=OH	3	AMM	[82]
**98**	dihydroxy-dimethoxy DHIF-Hex	R_3_=β-D-OGlcp R_2_=R_7_=OMe R_8_=OH	3	AMM	[82]
**99**	dihydroxy-trimethoxy DHIF-Pen	R_3_=β-D-OGlcp R_2_=R_7_=R_6_=OMe R_8_=OH	3	AMM	[82]
**100**	trihydroxy-dimethoxy DHIF-Hex	R_3_=β-D-OGlcp R_2_=R_6_=OMe R_8_=R_7_=OH	3	AMM	[82]
**101**	methylnissolin	R_1_=R_2_=R_3_=OMe	4	AMM, AM	[68,86]
**102**	(-)-methylnissolin3-O-β-D-glucoside	R_1_=β-D-OGlcp R_2_=OMe R_3_=OMe	4	AMM, AM	[68,81]
**103**	(-)-methylinissolin3-O-β-d-(6′-acetyl)-glucoside	R_1_= (6″-acetxyl)-β-D-OGlcp R_2_=R_3_=OMe	4	AMM, AM	[68,77]
**104**	maakiain	R_1_=OH R_3_=R_4_=OCH_2_O	4	AMM, AM	[76]
**105**	(6aR,11aR)-3,9,10-trimethoxypterocarpan	R_1_=R_2_=R_3_=OMe	4	AMM, AM	[76]
**106**	(6aR,11aR)-3-OH-9,10-dimethoxypterocarpan-3-O-β-D-glucopyranoside	R_1_=β-D-OGlcp R_2_=R_3_=OMe	4	AMM, AM	[76]
**107**	3,9-di-O-methylnissolin	R_1_=OH R_2_=R_3_=OMe	5	AMM	[87]
**108**	wogonin	R_1_=OMe R_2_=R_4_=OH	5	AMM	[78]
**109**	astraflavonoids A	R_2_=R_4_=R_6_=OH R_8_=β-D-OGlcp-(2→1)-(5‘-R)-Api	5	AMM	[69]
**110**	3-Hydroxy-9,10-dimethoxy pterocarpan	R_1_=OH R_2_=R_3_=OMe	5	AMM	[82]
**111**	9,10-dimethoxypterocarpan-3-O-glucoside	R_1_=β-D-OGlcp R_2_=R_3_=OMe	5	AMM	[82]
**112**	10-Hydroxy-3,9-dimethoxypterocarpan	R_1_=R_3_=OMe R_2_=OH	5	AMM	[82]
**113**	3-Hydro-9-MP-Hex-Hex	R_1_=β-D-OGlcp-Glcp R_3_=OMe	5	AMM	[82]
**114**	3-Hydro-9-MP-Hex	R_1_=β-D-OGlcp R_3_=OMe	5	AMM	[82]
**115**	3-Hydro-9,10-diMP-Pen-HeX	R_1_=OH-Pen-Glcp R_2_=R_3_=OMe	5	AMM	[82]
**116**	3-Hydro-9-MP-malonyl-Glc	R_1_=β-D-OGlcp-Mal R_3_=OMe	5	AMM	[82]
**117**	MP	R_3_=OMe	5	AMM	[82]
**118**	9,10-DiMP-3-O-malonyl-Glc	R_1_=OGlcp-Mal R_2_=R_3_=OMe	5	AMM	[82]
**119**	9,10-DiMP-3-O-acetyl-Glc	R_1_=β-D-OGlcp-Ac R_2_=R_3_=OMe	5	AMM	[82]
**120**	9,10-dimethoxypterocarpan-3-O-glucopyranoside	R_1_=β-D-OGlcp R_2_=R_3_=OMe	5	AMM	[82]
**121**	(-)-methylinissolin3-O-β-d-(6′-(E)-But-2-enoyl)-glucoside	R_1_=β-[6-(E)-But-2-enoyl]- D-OGlcp R_2_=R_3_=OMe	5	AM	[68]
**122**	(+)-vesticarpan	R_1_=R_2_=OH R_3_=OMe	5	AM	[68]
**123**	licoagroside D	R_1_=β-D-OGlcp R_2_=OH R_3_=OMe	5	AM	[68]
**124**	(6aR,11aR)-10-OH-3,9, -dimethoxypterocarpan	R_1_=R_3_=OMe R_2_=OH	5	AM	[76]
**125**	(-)-Methylinissolin 3-O-(6-acety1-β-D-glucopyranoside)	R_1_=O-(6-O-Ac-β-D-Glcp) R_2_=R_3_=OMe	5	AM	[76]
**126**	(-)-Methylinissolin 3-O-[6-O-(E)-but-2-enoyl-β-D-glucopyranoside]	R_1_=O-[6-O-(E)-but-2-enoyl-β-D-Glc] R_2_=R_3_=OMe	5	AM	[76]
**127**	2′,4′,4-trihydroxy-chaleone (Isoliquiritigenin)	R_1_=R_2_=R_3_=R_6_=OH	6	AMM, AM	[70,88]
**128**	4,4′,6′-trihydroxychalcone	R_2_=R_3_=R_6_=OH	6	AMM	[89]
**129**	2′-methoxyisoliquiritigenin	R_1_=OMe R_2_=R_6_=OH	6	AM	[70]
**130**	echinatin	R_4_=OMe R_2_=R_6_=OH	6	AM	[70]
**131**	licochalcone B	R_2_=R_6_=R_5_=OH R_4_=OMe	6	AM	[70]
**132**	4,4′-dimethyl-6′-hydroxychalcone	R_2_=R_4_=CH_3_ R_1_=H	6	AMM	[89]
**133**	4-methoxy-4′,6′-dihydroxychalcone	R_2_=R_1_=OH R_6_=OMe	6	AMM	[89]
**134**	4′-hydroxyflavonone-7-O-β-D-glucoside	R_2_=β-D-OGlcp R_8_=OH	7	AMM	[89]
**135**	naringin	R_4_=R_8_=OH R_2_=β-D-O-Glcp-α-L-Rha	7	AMM	[82]
**136**	3′,4′,7-trihydroxyflavone	R_2_=R_8_=R_9_=OH	7	AM	[70]
**137**	liquiritigenin	R_2_=R_8_=OH	7	AMM, AM	[70,89]
**138**	dihydroxyflavone	R_1_=R_2_=OH	7	AMM	[82]
**139**	isomucronulatol-7-O-glycoside	R_2_=β-D-OGlcp R_6_=OH R_7_=R_8_=OMe	8	AMM, AM	[68]
**140**	6″-O-acetyl-(3R)-7,2′-dihydroxy-3′,4′-dimethoxyisoflavan-7-O-β-D-glucopyranoside	R_2_=(6″-acetxyl)-β-D-OGlcp R_9_=R_8_=OMe	8	AMM	[77]
**141**	3,2′-dihydroxy-3′,4′-dimethylisoflavan-7-O-β-d-glucoside	R_2_=β-D-OGlcp R_8_=OMe R_9_=OMe R_10_=OH	8	AMM	[89]
**142**	astraflavonoids C	R_2_=R_10_=OH R_9_=R_7_=OMe R_8_=β-D-OGlcp	8	AMM	[69]
**143**	7-O-methylisomucronulatol	R_2_=R_7_=R_8_=OMe	8	AMM	[87]
**144**	5-hydroxyisomucronulatol 2′,5′-di-O-glucoside	R_2_=OH R_9_=R_6_=β-D-OGlcp R_8_=R_7_=OMe	8	AMM	[87]
**145**	astraisoflavanin	R_2_=β-D-OGlcp R_5_=R_6_=OMe R_7_=OH	8	AMM	[75]
**146**	3′-OH-2,4′-dimethoxyisoflavane-6-O-glc	R_9_=OH R_8_=R_11_=OMe R_3_=β-D-OGlcp	8	AMM	[90]
**147**	(3R)-isomucronulatol	R_2_=R_10_=OH R_8_=R_9_=OMe	8	AMM	[76]
**148**	isomucronulatol 5′-OH-2′,5′-di-O-glc	R_2_=OH R_6_=R_9_=β-D-OGlcp R_8_=R_7_=OMe	8	AMM	[76]
**149**	isomucronulatol 7,2′-di-O-β-glucoside	R_2_=R_6_=β-D-OGlcp R_8_=R_7_=OMe	8	AMM	[76]
**150**	Astraisoflavan 7-O-(6-malony1-β-D-glucopyranoside)	R_2_= O-(6-O-malonyl-β-D-Glcp) R_10_=OH R_8_=R_9_=OMe	8	AMM	[76]
**151**	7,2′-Dihydroxy-3′4′-dimethoxyisoflavan	R_2_=R_10_=OH R_9_=R_8_=OMe	8	AMM	[82]
**152**	2′-Hydroxy-3′,4′-dimethoxyisoflavan-7-O-Glc	R_2_=β-D-OGlcp R_10_=OH R_8_=R_9_=OMe	8	AMM	[82]
**153**	7-Hydroxy-6,4′-dimethoxyisoflavan	R_2_=OH R_3_=R_8_=OMe	8	AMM	[82]
**154**	trihydroxy-methoxyisoflavan-Hex-hex	R_10_=OMe R_3_=R_9_=OH R_8_=OGlcp-Glcp	8	AMM	[82]
**155**	trihydroxy-dimethoxyisoflavan-HeX	R_9_=R_10_=OMe R_8_=OGlc R_3_=R_7_=OH	8	AMM	[82]
**156**	isomucronulatol-Hex-Hex	R_2_=β-D-OGlc-Glc R_10_=OH R_5_=R_9_=OMe	8	AMM	[82]
**157**	dihydroxy-dimethoxyisoflavan	R_9_=R_10_=OMe R_3_=R_8_=OH	8	AMM	[82]
**158**	isomucronulatol-acetyl-Glc	R_2_=β-D-OGlc-Ac R_10_=OH R_8_=R_9_=OMe	8	AMM	[82]
**159**	3-Mucronulatol-O-glucopyranoside	R_2_=β-D-OGlcp R_9_=OH R_3_=R_8_=OMe	8	AMM	[82]
**160**	5′-Hydroxy-isomucronulatol-2′,5′-glucoside	R_3_=R_8_=β-D-OGlcp	8	AMM	[82]
**161**	2′,4′-Dimethoxy-3′-hydroxyisoflavan-6-O-Glc	R_10_=R_8_=OMe R_9_=OH R_3_=β-D-OGlcp	8	AMM	[82]
**162**	3,2′-Dihydroxy-3′,4′-dimethoxyisoflavan-7-O-Glc	R_2_=β-D-OGlcp R_10_=OH R_8_=R_9_=OMe	8	AMM	[82]
**163**	sphaerophyside SB	R_3_=OH R_1_=R_2_=OMe R_3_=β-D-OGlcp	9	AM	[67]
**164**	sophorophenolone	-	10	AM	[91]
**165**	(3R)-7,2′,3′-trihydroxy-4′-methoxy-isoflavane	R_1_=R_3_=R_4_=OH R_5_=OMe	12	AM	[92]
**166**	Trifolinhizin	R_1_=β-D-OGlcp	11	AMM	[93]
**167**	(3R)-(5′-hydroxy-2′,3′,4′-trimethoxyphenyl)-chroman-7-ol	R_1_=OH R_3_=R_4_=R_5_=OMe	12	AM	[68]
**168**	(3R)-8,2′-dihydroxy-7,4′-dimethoxyisoflavan	R_1_=OH R_2_=R_6_=OMe R_8_=OAc	12	AMM, AM	[94]
**169**	isomucronulatol 7,3′-di-O-glc	R_1_=R_4_=β-D-OGlcp R_3_=OH R_5_=OMe	12	AM	[76]
**170**	(3R)-8,2′-Dihydroxy-7,4′-dimethoxyisoflavan	R_1_=R_6_=OH R_2_=R_8_=OMe	13	AMM, AM	[95]
**171**	isomucronulatol	R_2_=R_7_=R_8_=OMe R_6_=OH	13	AMM, AM	[87]
**172**	7-O-methylisomucronulatol	R_2_= R_7_=R_8_=OMe R_6_=OH	13	AMM, AM	[96]
**174**	(3R)-7,2′,3′-Trihydroxy-4′-methoxy-isoflavane	R_2_=R_6_=R_7_=R_9_=OH R_8_=OMe	13	AM	[95]
**175**	(R)-3-(5-Hydroxy-2,3,4-trimethoxyphenyl)-chroman-7-ol	R_2_=R_9_=OH R_6_=R_7_=R_8_=OMe	13	AM	[68]
**176**	(3R)-(-)-Mucronulatol 7-O-β-D-glucoside	R_1_=β-D-OGlcp R_6_=R_8_=OMe R_7_=OH	13	AMM	[97]
**177**	6″-O-Acetyl-(3 R)-2′-hydroxy-3′,4′-dimethoyl-isoflavan 7-O-β-D- glucopyranoside	R_3_=β-D-6-O-Ac-Glcp R_6_=OH R_7_=R_8_=OMe	13	AMM	[98]
**178**	3′-Hydroxy-2′,4′-dimethoxyisoflavan 6-O-β-D-glucopyranoside	R_3_=β-D-OGlcp R_5_=R_7_=OH R_6_=R_8_=OMe	13	AMM	[99]
**179**	Astraflavonoid C	R_2_=R_6_=OH R_7_=R_9_=OMe R_8_=β-D-OGlcp	13	AMM	[69]
**180**	3,2′-Dihydroxyl-3′,4′-methoxyisoflavanone 7-O-β-D-glucoside	R_2_=β-D-OGlcp R_4_=R_6_=OH R_7_=R_8_=OMe	13	AMM	[100]
**181**	(3R,4R)-4,7-Hydroxy-2′,3′-dimethoxyisoflavane 4′-O-β-D-glucoside	R_2_=R_4_=OH R_5_=Me R_6_=β-D-OGlcp R_7_=R_8_=OMe	13	AMM	[101]
**182**	2′,5′-Dicarbonyl-3′,4′-dimethoxyisoflavanequinone 7-O-β-D-glucoside	R_1_=β-D-OGlcp	14	AMM	[93]
**183**	Pendulone	R_1_=OH	14	AM	[68]

Note: Unmarked in the table: R=H, Glc-glucose, Rha-rhamnose, Me-methyl, Ac-acetyl, Xyl-xylose, Glcp-glucopyranoside.

### 5.2. Saponins

**Figure 4:**
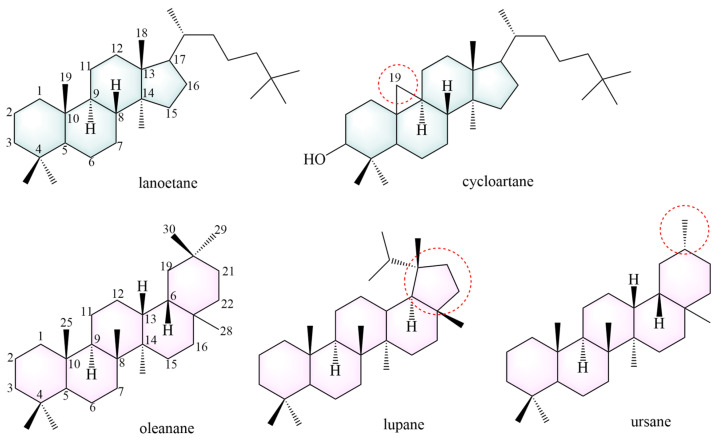
The structural backbones of saponins. The red circle indicates the site of modification in the saponins’s parent nucleus.

To date, 101 saponins have been isolated from AMM and AM. AMM contains 54 saponins, and AM contains 60 saponins, including 13 common saponins. Tetracyclic triterpenes and pentacyclic triterpenes are included, with tetracyclic triterpenes mainly comprising lanostane and cycloartane (Figure 4).

Most tetracyclic triterpenes possess the fundamental skeleton of a cyclopentane-perhydrophenanthrene ring system. Lanostane is formed via a chair-boat-chair-boat conformational cyclization of epoxy squalene. It exhibits anti-diabetic, neuroprotective [102], anti-tumor [103], anti-malarial [104], anti-aging [105], and anti-inflammatory effects [106]. The parent structural backbone of cycloartane is similar to that of lanostane, and dehydrogenation at C19 and C9 of cycloartane results in a three-membered ring. Cycloartane exerts neuroprotective and antioxidant effects [107].

**Figure 5:**
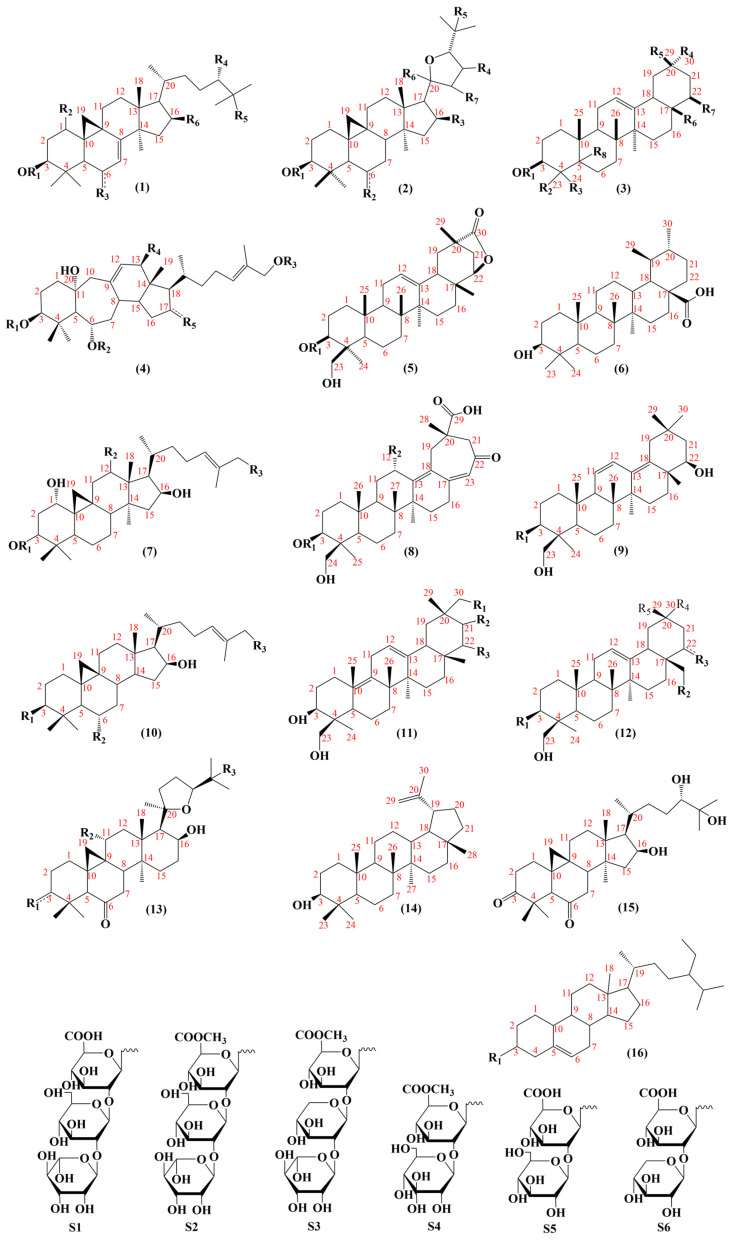
The structural backbones of saponins in AM and AMM.

Pentacyclic triterpenoids mainly include oleanane (**234**–**249**, **259**–**261**, **269**, **270**–**276**), ursane (**262**) and lupane (**281**). These constituents exhibit anti-cancer [108] and anti-inflammatory effects [109]. The structures of the saponin skeletons from AM and AMM are illustrated in Figure 5. The saponins were isolated from AMM and AM (Table 5).

Oleanane, also known as β-amyrane, has a basic structural backbone of polyhydropinene [40], and exhibits anti-inflammatory and hepatoprotective effects. The C19 and C21 positions of lupane form a five-membered ring. Lupane possesses antibacterial [110], cardioprotective [111], and anti-inflammatory effects [112]. Ursane, also known as α-amyrin, differs from oleanolic acid in that the C19 and C20 positions each contain a methyl group. Studies have indicated that ursane has anti-tumor [113] and neuroprotective effects [114].

**Table 5:** Saponins isolated from AM and AMM.

No.		Name	Substituent	Skeletons	Species	References
**184**		mongholicoside A	R_1_=β-D-Glcp R_2_=R_3_=R_4_=R_5_=R_6_=OH	1	AMM	[115]
**185**		mongholicoside B	R_1_=β-D-Glcp R_2_=R_4_=R_5_=R_6_=OH R_3_=O	1	AMM	[115]
**186**		alexandroside I	R_1_=β-D-Glcp R_3_=R_4_=R_5_=R_6_=OH	1	AMM	[81]
**187**		agroastragaloside I	R_1_=(2′,3′-di-OAc)-β-D-Xyl R_3_=β-D-Glcp R_6_=OH	1	AM	[116]
**188**		agroastragaloside II	R_1_=β-(2′-OAc)-D-Xyl R_2_=β-D-OGlcp R_6_=OH	1	AM	[91]
**189**		agroastragaloside V	R_1_=β-(2′-OAc)-D-Xyl R_2_=β-D-OGlcp	1	AM	[117]
**190**		astramembranoside B	R_1_=β-(2′-OAc)-D-Xyl R_6_=OH	1	AM	[91]
**191**		cyclocanthoside A	R_1_=β-D-Xyl R_6_=OH	1	AM	[91]
**192**		cyclocanthoside E	R_1_=β-D-Xyl R_2_=β-D-OGlcp R_6_=OH	1	AM	[70]
**193**		agroastragaloside	R_1_=2′-O-Ac-β-D-Xyl R_3_=β-D-OGlcp R_4_=R_5_=R_6_=OH	1	AM	[118]
**194**		huangqiyenin II	R_3_=O R_4_=R_5_=R_6_=OH	1	AM	[118]
**195**		huangqiyenin B	R_2_=β-D-Glcp R_3_=O R_4_=R_5_=R_6_=OH	1	AM	[118]
**196**		isocyclocanthoside E	R_1_=β-D-Xyl R_3_=β-D-OGlcp R_4_=R_5_=R_6_=OH	1	AM	[118]
**197**		aleksandroside I	R_1_=β-D-Glcp R_2_=H R_3_=R_4_=R_5_=R_6_=OH	1	AMM	[119]
**198**		astragaloside I	R_1_=(2′,3′-di-OAc)-β-D-Xyl R_2_=β-D-Glcp R_3_=R_5_=OH R_6_=Me	2	AMM, AM	[66,71]
**199**		astragaloside II	R_1_=(2′-OAc)-β-D-Xyl R_2_=β-D-Glcp R_3_=R_5_=OH R_6_=Me	2	AMM AM	[66,71]
**200**		astragaloside III	R_1_=β-D-Xyl-(2→1)-β-D-Glcp R_2_=R_3_=R_5_=OH R_6_=Me	2	AMM, AM	[66,71]
**201**		astragaloside IV	R_1_=β-D-Xyl R_2_=β-D-Glcp R_3_=R_5_=OH R_6_=Me	2	AMM, AM	[68,88]
**202**		isoastragaloside I	R_1_=R_2_=β-D-Glcp R_3_=R_5_=OH	2	AMM, AM	[65,90]
**203**		isoastragaloside II	R_1_=β-D-Xyl R_2_=β-D-Glcp R_3_=R_5_=OH R_6_=Me	2	AMM, AM	[74,120]
**204**		acetylastragaloside Ι	R_1_=(3′-OAc)-Xyl R_2_=β-D-Glcp	2	AMM, AM	[65,121]
**205**		astragaloside VII	R_1_=β-D-Xyl R_2_=R_5_=β-D-Glcp	2	AMM, AM	[65,86]
**206**		isoastragaloside VII	R_3_=R_5_=OH R_6_=Me	2	AMM, AM	[118]
**207**		agroastragaloside III	R_1_=(2′,3′-di-OAc)-β-D-Xyl R_2_=R_5_=β-D-OGlcp R_3_=R_5_=OH R_6_=Me	2	AM	[122]
**208**		agroastragaloside IV	R_1_=(2′-OAc)-β-D-Xyl R_2_=R_5_=β-D-OGlcp R_3_=R_5_=OH R_6_=Me	2	AM	[122]
**209**		astragaloside V	R_1_=β-D-Xyl-(2→1)-Glc R_5_=β-D-OGlcp R_3_=R_2_=OH R_6_=Me	2	AM	[123]
**210**		astragaloside VI	R_1_=β-D-Xyl-(2→1)-Glcp R_2_=β-D-OGlcp R_3_=R_5_=OH R_6_=Me	2	AM	[123]
**211**		astramembranoside A	R_1_=R_5_=β-D-OGlcp R_3=_OH R_6_=Me	2	AM	[91]
**212**		brachyoside B	R_2_=β-D-OGlcp R_3_=R_5_=OH R_6_=Me	2	AM	[91]
**213**		cycloastragenol	R_2_=R_3_=R_5_=OH R_6_=Me	2	AM	[123]
**214**		isoastragaloside IV	R_1_=β-D-Xyl R_5_=β-D-OGlcp	2	AM	[124]
**215**		astramembranin II	R_1_=β-D-Xyl R_2_=R_3_=R_5_=OH R_6_=Me	2	AM	[125]
**216**		huangqiyiesaponin C	R_1_=β-D-Glcp	2	AM	[126]
**217**		cyclounifolioside B	R_1_=β-D-Xyl-(2→1)-β-D-Glcp	2	AM	[91]
**218**		astraverrucin I	R_1_=α-L-Rha-(1→4)-β-D-Glcp R_2_=R_3_=R_5_=OH R_6_=Me	2	AM	[118]
**219**		isoastragaloside V	R_1_=Ara-(1→2)-β-D-Xyl R_2_=β-D-OXyl R_3_=R_5_=OH R_6_=Me	2	AM	[118]
**220**		neoastragaloside I	R_1_=2′,3′-O-di-Ac-β-D-Xyl R_2_=OH R_3_=R_5_=OH R_6_=Me	2	AM	[118]
**221**		huangqiyenin A	R_1_=β-D-Glcp R_3_=R_5_=OH R_6_=Me	2	AM	[118]
**222**		astrolanosaponin A2	R_1_=2-O-Ac-β-D-Glcp R_2_=H R_3_=OH R_5_=β-D-OGlcp R_6_=Me	2	AMM	[119]
**223**		cycloaraloside E	R_1_=β-D-Glcp R_2_=O R_3_=OH R_5_=β-D-OGlcp R_6_=Me	2	AMM	[119]
**224**		astrolanosaponin A1	R_1_=β-D-Glcp R_2_=β-D-OGlcp R_2_=OH R_3_=R_5_=OH R_4_=H R_6_=Me	2	AMM	[119]
**225**		astraverrucin II	R_1_=2-O-Ac-β-D-Glcp R_2_=OH R_3_=R_5_=OH R_6_=Me	2	AMM	[119]
**226**		huangqiyenin K	R_1_=β-D-Xyl R_2_=OAc R_3_=R_5_=OH R_6_=Me	2	AM	[127]
**227**		astramembrannin II	R_1_=Glcp R_3_=R_5_=OH R_6_=Me	2	AMM, AM	[128]
**228**		agroastragaloside III	R_1_=β-D-Xyl R_3_=R_5_=OH R_6_=Me	2	AM	[41]
**229**		agroastragaloside IV	R_1_=2-O-Ac-β-D-Xyl R_2_=R_5_=β-D-OGlcp R_3_=OH R_6_=Me	2	AM	[41]
**230**		isoastragaloside I	R_1_=2,4-O-Ac2-β-D-Xyl R_2_=β-D-Glcp R_3_=R_5_=OH R_6_=Me	2	AMM, AM	[100]
	**231**	astrolanosaponin B	R_1_=β-D-Glcp R_2_=β-D-OGlcp R_2_=O R_3_=OH R_6_=Me	2	AMM	[129]
**232**		astrolanosaponin D	R_1_=β-D-Glcp R_2_=OH R_3_=R_5_=OH R_6_=Me	2	AMM	[119]
**233**		astrolanosaponin E	R_1_=β-D-Glcp R_2_=OH R_3_=R_5_=OH R_6_=Me	2	AMM	[119]
**234**		soyasapogenol B	R_7_=OH R_3_=Me R3=R4=R5=R6=Me	3	AM	[123]
**235**		soyasapogenol B	R_7_=OH R_3_=MeOH R_3_=R_4_=R_5_=R_6_=Me R_8_=Me	3	AM	[118]
**236**		astraisoolesaponins A	R_1_=S1 R_7_=O R_2_=MeOH R_3_=R_4_=R_5_=R_6_=Me	3	AMM	[118]
**237**		astraisoolesaponins B	R_1_=S1 R_2_=R_5_=MeOH R_7_=OH R_6_=R_3_=R_4_=Me	3	AMM	[118]
**238**		astraisoolesaponins Cl	R_1_=S4 R_2_=MeOH R_7_=OH R_3_=R_5_=R_6_=Me R_4_=COOH	3	AMM	[118]
**239**		astraisoolesaponins C2	R_1_=S2 R_2_=MeOH R_7_=OH R_3_=R_5_=R_6_=Me R_4_=COOH	3	AMM	[118]
**240**		astraisoolesaponins E1	R_1_=S5 R_2_=R_6_=MeOH R_7_=O R_4_=COOH R_3_=R_5_=Me	3	AMM	[118]
**241**		astraisoolesaponins E2	R_1_=S6 R_2_=R_6_=MeOH R_7_=O R_4_=COOH R_3_=R_5_=Me	3	AMM	[118]
**242**		azukisaponin V	R_1_=S1 R_2_=MeOH R_3_=R_4_=R_5_=R_6_=Me R_7_=OH	3	AMM	[118]
**243**		astragaloside VIII methyl ester	R_1_=S3 R_2_=MeOH R_7_=OH R_3_=R_4_=R_5_=R_6_=Me	3	AMM	[118]
**244**		robinioside F	R_1_=S1 R_2_=MeOH R_7_=OH R_3_=R_5_=R_6_=Me R_4_=CH_2_OH	3	AMM	[118]
**245**		robinioside B	R_1_=S1 R_2_=MeOH R_7_=OH R_3_=R_5_=R_6_=Me R_4_=COOH	3	AMM	[118]
**246**		cloversaponin III	R_1_=S5 R_2_=MeOH R_7_=O R_3_=R_5_=R_6_=Me R_4_=COOH	3	AMM	[118]
**247**		soyasaponin I	R_1_=β-D-GlcA-(2→1)-β-D-Xyl-(2→1)-α-L-Rha R_2_=CH_2_OH R_3=_R_4_=R_5_=R_6_=Me R_7_=OH	3	AMM, AM	[66,71]
**248**		astragaloside VIII	R_1_=β-D-GlcA-(2→1)-β-D-Xyl-(2→1)-α-L-Rha R_2_=CH_2_OH R_3=_R_4_=R_5_=R_6_=Me R_7_=OH	3	AMM, AM	[71,120]
**249**		robinioside F	R_1_=β-D-OGlcA-(1→2)-β-D-Glcp-(1→2)-α-L-Rha R_2_=R_4_=MeOH R_3_=R_5_=R_6_=MeOH R_7_=OH	3	AMM	[129]
**250**		huangqiyenin E	R_1_=R_2_=Ac R_4_=OAc R_5_=OH R_3_=β-D-Glcp	4	AM	[130]
**251**		huangqiyenin O	R_1_=R_2_=R_4_=R_5_=OH R_3_=β-D-Glcp	4	AM	[130]
**252**		huangqiyegenin III	R_1_=R_2_=Ac R_3_=OAc	4	AM	[130]
**253**		huangqiyegenin IV	R_1_=R_2_=Ac	4	AM	[130]
**254**		trideacetylhuangqiyegenin III	R_3_=OH	4	AM	[130]
**255**		huangqiyenin G	R_1_=H R_2_=Ac R_3_=β-D-Glcp R_4_=O R_5_=OH	4	AM	[131]
**256**		huangqiyenin W	R_1_=H R_2_=R_4_=OAc R_3_=β-D-Glcp	4	AM	[131]
**257**		huangqiyenin R	R_1_=R_2_=H R_5_=OH R_4_=OAc R_3_=β-D-Glcp	4	AM	[131]
**258**		huangqiyenin Q	R_1_=Ac R_4_=R_5_=OH R_3_=β-D-Glcp	4	AM	[131]
**259**		astraisoolesaponins D	R_1_=S1	5	AMM	[118]
**260**		astraisoolesaponins F	R_1_=S2	5	AMM	[118]
**261**		astroolesaponin D	R_1_=α-L-Rha-(1→2)-β-D-Glcp-(1→2)-β-D-GIcA	5	AMM	[41]
**262**		ursolic Acid	-	6	AMM	[86]
**263**		Mongholicoside I	R_3_=β-D-OGlcp	7	AMM	[41]
**264**		Mongholicoside II	R_1_=Ac R_2_=OH R_3_=β-D-OGlcp	7	AMM	[41]
**265**		astraisoolesaponin A1	R_1_=α-L-Rha-(1→2)-β-D-Glcp-(1→+2)-β-D-Glcp R_2_=OH	8	AMM	[41]
**266**		astraisoolesaponin A2	R_1_=β-D-Xyl-(1→2)-β-D-GlcA R_2_=OH	8	AMM	[41]
**267**		astraisoolesaponin A3	R_1_=β-D-Glcp-(1→2)-β-D-GlcA R_2_=OH	8	AMM	[41]
**268**		astroolesaponin F	R_1_=β-D-OGlcA-OMe-(1→2)-β-D-Glcp-(1→2)-α-L-Rha	9	AMM	[129]
**269**		huangqiyenin L	R_1_=β-D-OXyl R_2_=OAc R_3_=β-D-OGlcp	10	AM	[127]
**270**		(3β,21α)-olean-12-ene-3,21,24-triol	R_2_=OH	11	AM	[127]
**271**		(3β,22β)-olean-12-ene- 3,22,24,29-tetro	R_1_=R_3_=OH	11	AM	[129]
**272**		soyasapogenol E	R_3_=O	11	AM	[127]
**273**		astroolesaponin C1	R_1_=OS4 R_2_=OH R_3_=OH R_4_=COOH	12	AMM	[129]
**274**		astroolesaponin C2	R_1_=β-D-OGlcA-OMe-(1→2)-β-D-Glcp-(1→2)-α-L-Rha R_2_=OH R_3_=OH R_4_=COOH	12	AMM	[129]
**275**		robinioside B	R_1_=OS1 R_2_=OH R_3_=OH	12	AMM	[129]
**276**		astroolesaponin A	R_1_=β-D-OGlcp-(1→2)-β-D-Glcp-(1→2)-α-L-Rha R_4_=R_5_=Me	12	AMM	[129]
**277**		astrolanosaponin C	R_1_=β-D-OGlcp R_3_=OH	13	AMM	[119]
**278**		cyclocephaloside II	R_1_=4-O-Ac-β-D-Xyl R_3_=OH	13	AM	[68]
**279**		huangqiyegenin V	R_1_=O R_2_=R_3_=OH	13	AM	[127]
**280**		huangqiyegenin I	R_1_=R3=OH R_2_=OH	13	AM	[127]
**281**		lupeol	-	14	AMM	[97]
**282**		huangqiyegenin VI	-	15	AM	[127]
**283**		β-daucosterol	R_1_=OH	16	AMM	[66]
**284**		d-3-O-methyl-chiro-inositol	R_1_=β-D-OGlcp	16	AMM	[66]

Note: Unmarked in the table: R=H, Glc-glucose, Rha-rhamnose, Me-methyl, Ac-acetyl, Xyl-xylose, OGlcA-glucuronic acid, Glcp-glucopyranoside.

### 5.3. Others Constituents

**Figure 6:**
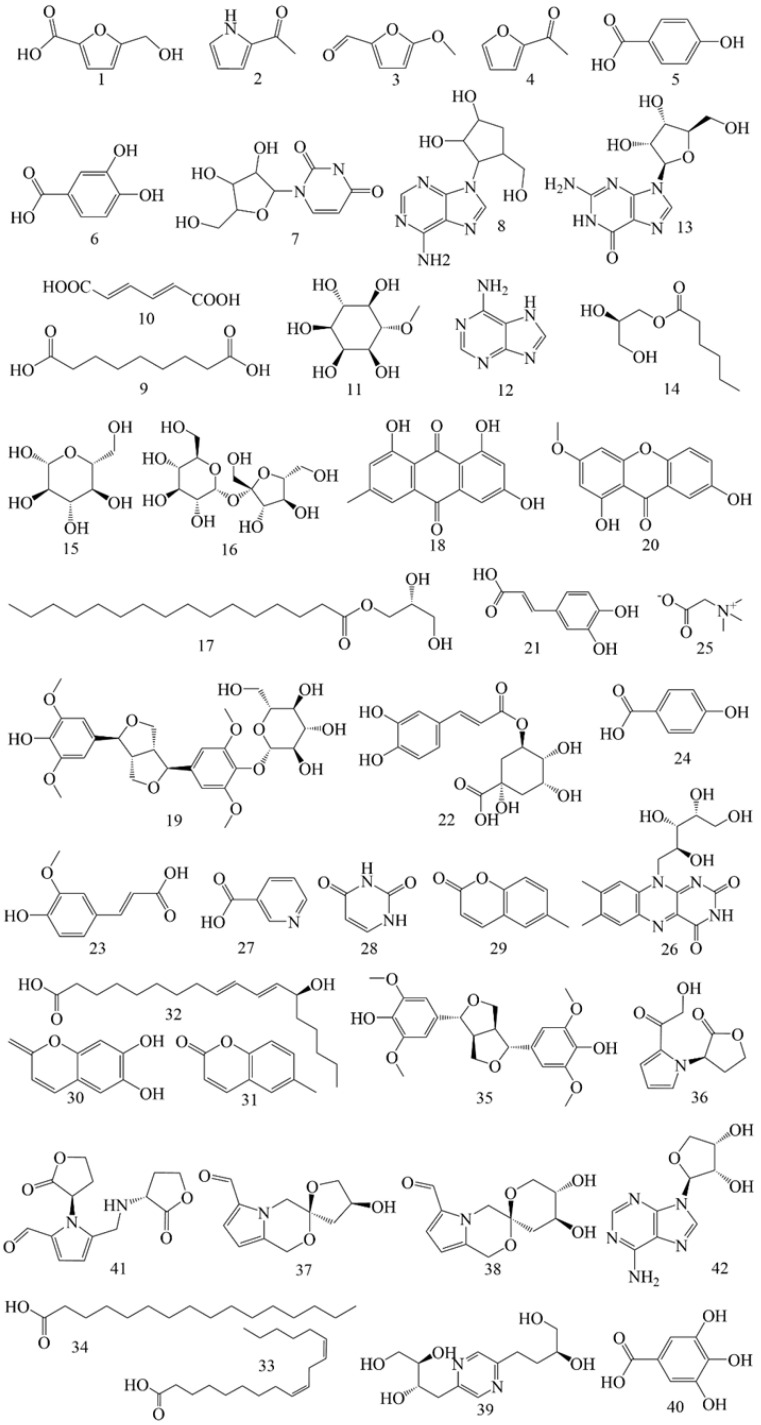
The structural backbones of s other structures in AM and AMM.

In addition to flavonoids and saponins, AR contains numerous other constituents. A total of 40 additional constituents have been reported in AMM and AM (Table 6), including 18 components in AMM and 24 in AM. The structures of these constituents from AM and AMM are shown in Figure 6.

Alkaloids (**286**, **291**–**292**, **296**–**297**, **340**–**342**) exhibit anti-inflammatory [132], neuroprotective [133] and antiviral properties [134,135].

Glycosides (**299**–**300**) are among the primary metabolites of AR, playing a role in immune regulation and exhibiting anti-inflammatory properties [136,137]. Phenolics (**5**–**6**, **38**) efficiently scavenge free radicals [138] and exhibit antioxidant properties [139].

Quinones (**302**, **304**) are primarily classified into four categories: benzoquinones, naphthoquinones, phenanthraquinones and anthraquinones. These constituents demonstrate cardioprotective [140], anti-inflammatory [141], anti-tumor [142], and anti-oxidation effects [143] while also contributing to kidney protection [144].

Isocoumarins (**313**–**314**) are a class of constituents characterized by a benzopyranone structure [145]. Studies have shown that these compounds possess various pharmacological properties. Isocoumarin constituents have demonstrated antioxidant and anti-diabetic effects [136,146]. At the same time, isocoumarins and their glycosyl derivatives exhibit significant therapeutic potential against cancer by influencing several critical cellular processes, such as apoptosis, autophagy, and cell cycle regulation [135,147].

## 6. Pharmacological Studies

**Figure 7:**
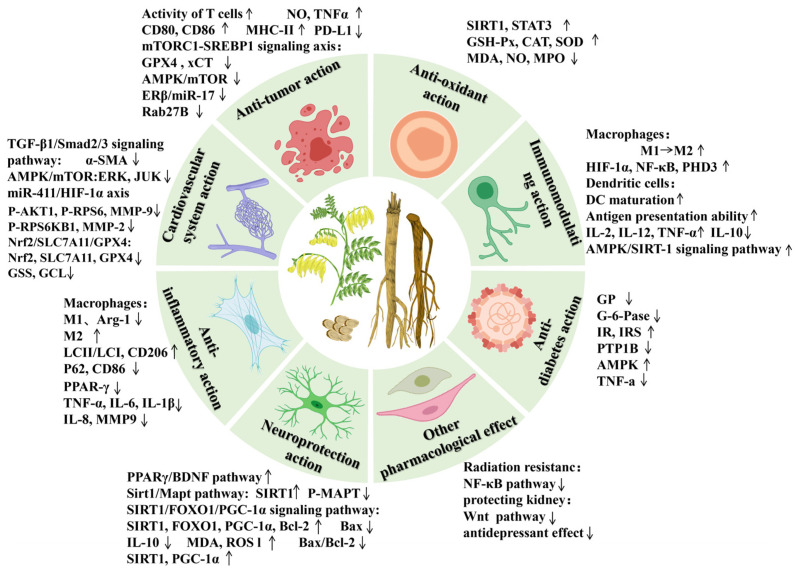
The modern pharmacologic actions and mechanisms of AR. The upward arrow denotes upregulation of the protein expression and activation of the signaling pathway, whereas the downward arrow represents downregulation of the protein and inhibition of the pathway.

Modern pharmacological studies have demonstrated that AR possesses various pharmacological effects, including anti-tumor, lowering blood glucose, cardiovascular and cerebrovascular protection, immune function improvement, anti-inflammatory, neuroprotection, protecting liver damage, anti-oxidative stress, and other biological activities [3,4,5,6,7,8,9] (Figure 7). The pharmacological effects of the active components of AR are shown in Table 7.

### 6.1. Anti-Tumor Action

AR, which is rich in Astragalus polysaccharides (APSs), flavonoids, saponins, and other active ingredients, can effectively inhibit tumor metastasis and diffusion.

Chronic inflammation is considered to be the cause of many diseases, such as tumors. APS can alleviate inflammation caused by lipopolysaccharide (LPS) and inhibit the inflammatory reaction of the tumor microenvironment in exosomes [148].

Numerous research studies have shown that dendritic cells (DCs) and T cells are modulators of immune checkpoint therapy and other tumor immunotherapies [149]. APS has gained recognition as an anti-tumor immunomodulator in clinical practice. APS can promote DC and T cell activation by increasing MHC-II, CD80, and CD86 expression [150]. It can regulate immune function and autophagy and enhance the efficacy of chemotherapeutic or targeted drugs by reducing their toxicity [3]. The combination of APS and 5-Fluorouracil can enhance the anti-tumor effect and reduce damage to the immune system [151]. Formononetin restored the activity of T cells and inhibited the growth of tumor xenografts [152].

Promoting autophagy and apoptosis in tumor cells can directly inhibit their growth of tumor cells [153]. Research findings suggest that APS can activate macrophages to release NO and TNF-α, which directly blocks cancer cell growth [154]. APS can also activate macrophages by inducing apoptosis, so that the cell cycle remains in the G2 phase, thereby inhibiting the growth of tumor cells [151]. Calycosin has been found to block the growth cycle of tumor cells, inhibit their proliferation of tumor cells, and induce apoptosis [155]. Formononetin has been shown to inhibit cell proliferation, tube formation, cell migration and promote tumor cell apoptosis by suppressing PD-L1 [152]. Studies have demonstrated that calycosin can induce autophagy and apoptosis in tumor cells by modulating the AMPK/mTOR, ERβ/miR-17, and Rab27B-dependent signaling pathways [156,157,158].

Ferroptosis has emerged as a promising approach for anti-tumor therapy, with targeting ferroptosis to eliminate tumor cells being recognized as a potentially effective strategy [159]. Formononetin triggers ferroptosis in tumor cells by modulating the mTORC1-SREBP1 signaling axis and suppressing the expression of key ferroptosis-related proteins, including GPX4 and xCT [160].

### 6.2. Antioxidant Action

At present, AR can resist oxidative stress by directly removing free radicals, improving the activity of antioxidant enzymes, inhibiting the activity of promoting enzymes, and regulating signaling pathways [4].

Oxidative stress elevates intracellular levels of reactive oxygen species (ROS), leading to cellular damage. Astragaloside IV (AS-IV) and formononetin have been shown to mitigate oxidative stress by activating the SIRT1 and STAT3 pathways, thereby exerting cytoprotective effects [161,162].

Formononetin, calycosin, and calycosin-7-glucoside demonstrated significant antioxidant activity, as evidenced by their free radical scavenging capabilities assessed through DPPH (2,2-diphenyl-1-picrylhydrazyl) radical scavenging activity and oxygen radical absorbance capacity (ORAC) assays [163].

Calycosin mitigates oxidative stress by suppressing the generation of reactive oxygen species (ROS) and enhancing the activity of antioxidant enzymes, including glutathione peroxidase (GSH-Px), catalase (CAT), and superoxide dismutase (SOD) [164]. APS enhanced the antioxidant capacity of tumor-bearing mice, specifically, APS reduced the levels of malondialdehyde (MDA), nitric oxide (NO), and myeloperoxidase (MPO), while increasing the activities of SOD, CAT, and GSH-Px by establishing a mouse ascites tumor model [165]. APS can mitigate oxidative stress and immune injury induced by acute cerebral ischemia-reperfusion injury in rats. This is achieved by enhancing SOD activity, increasing glutathione (GSH) levels, and reducing MDA content [166].

### 6.3. Cardiovascular System Action

AR has a protective effect on cardiovascular diseases because of its rich bioactive constituents, which exert various pharmacological actions that benefit the cardiovascular system [41,167].

The combination of Astragalus and Angelica has been shown to modulate the TGF-β1/Smad2/3 signaling pathway, thereby suppressing the expression of aortic α-SMA. This inhibition contributes to the prevention of vascular intima proliferation [168]. Calycosin can inhibit vascular calcification by inhibiting AMPK/mTOR [169]. AS-IV has been shown to promote neovascularization and protect cardiac function by modulating the miR-411/HIF-1α signaling axis [170].

Vascular remodeling is a common pathological process [171]. Research indicates that cycloastragenol downregulates the expression of P-AKT1, P-RPS6, and P-RPS6KB1 through the AKT1/RPS6KB1 signaling pathway, thereby enhancing myocardial autophagy. Simultaneously, it inhibits the expression of MMP-2 and MMP-9, improving cardiac insufficiency and remodeling [172]. Additionally, AS-IV has been shown to reduce CDK2 activity and block the G1/S phase transition, thereby mitigating pathological vascular remodeling in atherosclerosis [173].

AS-IV significantly inhibits the phosphorylation of ERK, JNK, and p38 MAPK in endothelial cells, as well as the phosphorylation of the upstream regulator TAK1. This inhibition suppresses the proinflammatory activation of vascular endothelial cells, reduces monocyte migration and adhesion, and ultimately exerts a protective effect on cardiovascular health [174].

Calycosin upregulates the protein expression of Nrf2, SLC7A11, GPX4, GSS, and GCL by activating the Nrf2/SLC7A11/GPX4 signaling pathway. This enhancement strengthens the antioxidant capacity of the myocardial tissue and effectively inhibits ferroptosis in cardiomyocytes [175].

### 6.4. Immunomodulating Action

According to TCM theory, AR can tonify qi and elevate yang, which is closely associated with immune regulation in humans [6].

DCs are specialized antigen-presenting cells that initiate the primary immune response. APS promote DC maturation, enhance their antigen-presentation capabilities, and reduce their endocytic activity [176].

Macrophages play critical roles in both innate and adaptive immunity [177]. Under the influence of cytokines in the microenvironment, macrophages differentiate into various types of tumor-associated macrophages (TAMs), primarily M1 and M2 phenotypes [178]. AS-IV increases interferon expression and restores suppressed innate immune function by modulating the Cgas/STING signaling pathway [179]. Moreover, AS-IV stimulates the HIF-1α/NF-κB signaling pathway, leading to the upregulation of HIF-1α, NF-κB, and PHD3 protein expression, thereby augmenting macrophage immune function [180].

The immune response is a process in which immune substances generate specific effects. By effectively enhancing the body’s immune response, the stability of the internal environment can be maintained [50]. Treatment with APS promotes the secretion of IL-2, IL-12, and TNF-α in serum, while concurrently decreasing IL-10 levels, thereby enhancing immune responses [181]. Furthermore, APS activates the AMPK/SIRT-1 signaling pathway, thereby alleviating OTA-induced immune stress in both in vitro and in vivo models [182].

### 6.5. Anti-Inflammatory Action

It has been proven that AR has strong anti-inflammatory activity because the presence of various bioactive constituents in AR that exhibit anti-inflammatory effects.

Some active constituents of AR can directly inhibit the expression of related inflammatory factors. Formononetin inhibits the MAPK signaling pathway, upregulates peroxisome proliferator-activated receptor-γ (PPAR-γ) in the nucleus, and reduces the release of inflammatory factors, thereby exerting anti-inflammatory effects [183]. Research has shown that calycosin has a strong anti-inflammatory effect, which can reduce the levels of TNF-α, interleukin-6 (IL-6), and IL-1β [184]. Quercetin significantly downregulated TNF-α-induced MMP-9 expression in GES-1 cells via the TNFR-c-Src-ERK1/2, c-Fos, and NF-κB signaling pathways, demonstrating its potent anti-inflammatory effects [185,186]. Hou et al. (2019) demonstrated that the production of histamine and proinflammatory cytokines, including IL-6, interleukin-8 (IL-8), IL-1β, and TNF-α, was significantly reduced in KU812 cells treated with quercetin following inflammation. This finding provides evidence that quercetin possesses anti-inflammatory properties [187].

Macrophages are divided into M1 and M2 types [178,188]. M1 macrophages secrete an array of inflammatory mediators, including IL-1β, interferon-γ, and TNF-α, which exacerbate secondary injuries. Conversely, M2 macrophages secrete anti-inflammatory factors to mitigate inflammation in the affected area. Studies have shown that the ethanol extract of AR significantly inhibits the expression of Arginase-1, a marker of the M1 macrophage phenotype [189]. APS suppresses M1 macrophage expression, enhances M2 macrophage expression, facilitates the transition from the M1 to M2 phenotype, and ameliorates the inflammatory microenvironment in experimental autoimmune encephalomyelitis mice [190]. Formononetin increases the expression of LCII/LCI and CD206, decreases the expression of P62 and CD86, and mitigates inflammation by modulating macrophage autophagy and polarization [191,192].

### 6.6. Anti-Diabetes Action

The bioactive compounds in AR, including polysaccharides, flavonoids, and saponins. These constituents may exert their effects through multiple mechanisms to modulate the blood glucose levels.

One study demonstrated that AS-IV can lower blood glucose levels in high-fat diet plus streptozotocin-induced diabetic mice by inhibiting glycogen phosphorylase (GP) and glucose-6-phosphatase (G-6-Pase) activities, thereby suppressing hepatic glycogenolysis and glucose oxidation in the liver [193].

APS exhibits a potent ability to rectify the abnormally elevated protein tyrosine phosphatase 1B (PTP1B) activity in skeletal muscle during insulin resistance, thereby enhancing insulin receptor (IR) and insulin receptor substrate (IRS) tyrosine phosphorylation, ameliorating insulin signaling, and increasing insulin sensitivity [194]. Concurrently, APS can augment insulin sensitivity by activating protein kinase AMPK and promoting glucose uptake in adipocytes [195]. Additionally, the combination of APS with insulin has been shown to diminish the insulin resistance index by reducing TNF-α expression, which is beneficial for the management of diabetes [196].

### 6.7. Neuroprotection Action

AR and its active ingredients have been reported to show good curative effects in anti-nerve damage and protection of nerve function [197].

Glial cells and neurons are the two main cell types in the nervous system and play important roles in the repair of nerve injury [198]. AS-IV also protects against neuronal apoptosis by promoting the PPARγ/BDNF signaling pathway [199]. Moreover, it can upregulate SIRT1 through the Sirt1/Mapt pathway and further control MAPT modification to reduce the hyperphosphorylation of MAPT, thereby protecting against neuronal apoptosis [200]. Calycosin-7-O-β-D-glucopyranoside mitigates neuronal injury by modulating the SIRT1/FOXO1/PGC-1α pathway, enhancing the expression of SIRT1, FOXO1, PGC-1α, and Bcl-2, while repressing Bax expression [201].

Nerve injury is closely associated with neuroinflammation in the brain [202]. IL-10 expression in cortical neurons can be activated by formononetin to inhibit neuroinflammation and protect the nerves [203].

Similarly, neuronal injury is associated with oxidative stress in the brain [202]. Formononetin can also affect oxidative stress, reduce MDA and ROS levels, enhance mitochondrial membrane potential and cell viability, and improve nerve injury [58,204]. Additionally, formononetin has been shown to improve neurological deficits by stimulating the PI3K/Akt signaling pathway and downregulating the Bax/Bcl-2 ratio [205]. As a major active constituent of AR, calycosin-7-O-β-D-glucopyranoside alleviates neuronal injury by upregulating SIRT1 and PGC-1α protein expression and reducing excessive mitochondrial fission and overactivation of mitochondrial autophagy [206].

### 6.8. Other Pharmacological Effect

Moreover, AR can confer resistance to radiation, thereby safeguarding retinal ganglion cells, facilitating osteogenesis, and preserving kidney function [207]. a AR exerts a protective influence on the visceral organs by mitigating oxidative stress, reducing inflammation, and inhibiting visceral fibrosis and apoptosis [208]. Quercetin-3-O-β-d-glucopyranoside can significantly promote cell proliferation and promote osteogenesis [209]. A study indicated that APS elicits a pro-growth effect on bone marrow stromal cells (BMSCs) exposed to 2 Gy12C6^+^ radiation, which may be associated with the downregulation of NF-κB signaling pathway-related proteins and maintenance of genomic stability in BMSCs [210]. AR has a protective effect on rat retinal ganglion cells. Simultaneously, it inhibits the apoptosis of lower-glucose tubular epithelial cells [211,212]. Tao et al. (2016) developed a mouse model of depression and conducted behavioral experiments related to depression. The results revealed that both liquiritigenin (7.5 mg/kg and 15 mg/kg) and fluoxetine (20 mg/kg) markedly ameliorated depressive symptoms [213].

**Table 7:** Pharmacological effects of the active components of AR.

Constituents	Pharmacological Effect	Experimental Model	Concentration	References
Calycosin	Nervous system disease	HEK 293 cell	50 μM	[214]
	Estrogen-like effect	MCF-7 cell female kunming mice (weight: 18–22 g, age: 12 weeks)	8 μM in vitro 1, 2, 4 mg/kg in vivo	[215]
	Anti-inflammatory effects	HaCaT, NHEK cell male C57BL/6 mice (weight: 22.78 ± 0.85 g; age: 8 weeks)	0, 2, 5, 10 μM in vitro 5 mg/mL in vivo	[216]
	Antiviral effects	HUVEC, MDCK cell	20 μg/mL	[217]
	Anti-Oxidative	male Balb/C mice (weight: 20 ± 2 g, age: 8–10 weeks)	25, 50 mg/kg	[218]
	Breast cancer	MDA-MB-231 cell	10 μM	[219]
	Anti-fatty liver	male ICR mice	30, 60 mg/kg	[220]
	Cervical cancer	SiHa, CaSki, C-33A, HeLa, Etc1/E6E7 cell	50 μM	[221]
	Anti-osteosarcoma	U2OS cell	0, 10, 20, 40 μM	[222]
	Cancers of the liver	HepG2, Hep3, Huh7 cell	100 μM	[223]
	Diabetic nephropathy	NRK-52E cell	10 μg/mL	[224]
	Cardiovascular protection effect	male SD rat (weight: 240–260 g)	20 mg/kg	[225]
	Acute Lung Injury	MLE-12 cell male C57BL/6N mice (weight: 18–22 g, age: 8–10 weeks)	30 μg/mL in vivo 12.5 mg/kg in vitro	[226]
	Pancreatic cancer	PANC1, MIA PaCa-2, RAW 264.7, Pan02 cell	50 μM	[227]
Calycosin-7-O-β-D-glucopyranoside	Anti-Oxidative	BRL-3A cell	10, 20, 40 mg/L	[228]
	Anti-myocardial hypertrophy	male SD rat (weight: 247–250 g, age: 10 weeks)	26.8 mg/kg	[229]
	Neuronal Apoptosis	HT22 cell	15 μg/mL	[201]
	cervical cancer	-	-	[230]
	Immunosuppression	Inbred strain male, female Balb/c mice (age:6–8 weeks)	-	[231]
	ischemia-reperfusion injury	male Wistar rats	15, 30 mg/kg	[232]
	cervical cancer	HeLa cells	20, 40, 80 μg/mL	[233]
	osteoarthritis	Adolescen New Zealand white rabbit, 9 (weight: 3.0–3.5 kg)	200 μg/ ml	[234]
Formononetin	Anti-Oxidative	male SD rat (weight: 160–170 g)	10, 20, 40 mg/kg	[162]
	Anti-liver damage	male CD-1 mice (age: 7 years)	50, 100 mg/kg	[235]
	Anti-inflammatory effects	HaCaT cell, male BALB/c mice (age: 6–8 weeks)	0.1, 1, 10 μM in vitro 10 mg/kg in vivo	[236]
	Neuroprotection	male SD rat (weight: 160–180 g)	30 mg/kg	[237]
	Immunosuppression	Hep G2 cell	10, 50 mg/kg·	[238]
	Anti-tumor effects	CNE2 cell	10, 20, 40 μM	[239]
	Protecting heart muscle cells	male C57BL/6 mice (weight: 18.9 ± 1.0 g)	20, 40 mg/kg	[240],
	Protective effect on osteoblasts	ROB cell	10-6, 10-5, 10-4 mol/L	[241]
Ononin	Improve renal injury	male SD rat (weight: 250 ± 00 g)	50, 200 mg/kg	[242]
	Anti-inflammatory effects	RAW 264.7 cell	5, 25, 50, 100 μM	[243]
Isoquercitrin	Anti-Oxidative	PC12 cell	1, 10, 100 μmol/L	[244]
	Anti-inflammatory effects	KU812 cell	12.5, 25, 50 μg/mL	[245]
	Promoting osteogenesis	BMSC male Wistar rat (weight: 150 ± 10 g, age: 6 weeks)	0.1, 1 μM in vitro, 10mg/kg in vivo	[246]
	Anti-tumor effects	SD rat	-	[247]
	Diuretic effect	SH rat (weight: 250–300 g, age: 3–4 months)	10 mg/kg	[248]
	Anti-hypertension	male Wistar rat (weight: 250–300 g, age: 3–4 months)	2, 4 mg/kg	[249]
	Anti-liver damage	male kunming mice (weight: 20–25 g)	10, 20, 50 mg/kg	[250]
Isorhamnetin	Anti-tumor effects	AGS, MKN45, HFE-145 cell	0, 10, 25, 50 μm	[251]
	Anti-osteoporosis	SD rat (weight: 180 ± 20, age: 11 weeks)	30 mg/kg	[252]
	Anti-Oxidative	H9c2 cell	0, 3, 6, 12, 25, 50 μM	[253]
	Anti-inflammatory effects	HGFs cell	10, 20, 40 μM	[254]
Kaempferol	breast cancer	SK-BR-3 cell	30, 60 μmol/L	[255]
	Anti-inflammatory effects	PC12 cell	20, 40, 60, 80, 100 μmol/L	[256]
	Anti-liver damage	male Kunming mice (weight: 20–22 g)	6, 18mg/kg	[257]
Quercetin	Anti-Oxidative	Human endometrial stromal cell	10, 20 μmol/L	[258]
	Anti-liver damage	male Wistar rat (weight: 240 ± 20 g)	5, 10, 20 mg/kg	[259]
	Anti-inflammatory effects	male SD rat (weight: 250–300 g)	20 mg/kg	[260],
	Neuroprotection	SH-SY5Y cell	0.1, 1, 10, 25 μmol/L	[261]
	Anti-tumor effects	RPMI-8226, NCI-H929 cell	0, 0.01, 0.1, 1, 10, 50, 100 µM	[262]
	Heart protective effect	H9C2 cell male SD rat (weight: 180–200 g)	50 mg/kg in vivo, 50 μM in vitro	[263]
	Anti-aging	-	-	[264]
	Immunomodulating effects	male C57BL/6 mice (weight:20–22 g, age: 8 weeks)	50, 100 mg/kg	[265]
	Scavenging free radicals	-	-	[266]
Isoliquiritigenin	Anti-tumor effects	Human lung adenocarcinoma, HCC827, NCI-H1650, NCI-H1975, A549 cell, 293T, NIH3T3	10, 20, 40 µM	[267]
	Anti-Oxidative	female Swiss-Webster mice (age: 9 weeks)	0, 50, 100, 300 mg/kg	[268]
	Anti-inflammatory effects	THP-1 cells	0, 1, 3, 5, 7, 10 µM	[269]
Liquiritigenin	Anti-inflammatory effects	Swiss albino mice (weight: 180–200 g)	30, 100, 300 mg/kg	[270]
	Anti-tumor effects	H1299 cell	0.1, 0.2, 0.4, 0.8 mmol/L	[271]
	Anti-diabetes	male Swiss albino mice (weight: 25–30 g)	50, 100, 200 mg/kg	[272]
	romoting osteogenesis	MC3T3-E1 cell	0.04, 0.4, 4 µM	[273]
	Anti-depression	male ICR mice (weight: 20–22 g)	20, 7.5, 15 mg/kg	[213]
	Anti-liver fibrosis	male C57BL/6 mice (weight: 20–22 g)	10, 30 mg/kg	[274]
Pratensein	Improving cognitive impairment	male Wistar rat (weight: 300 ± 20 g, age: 10 weeks)	10, 20 mg/kg	[275]
Echinatin	Anti-tumor effects	KYSE 30, KYSE 270 cell, male nude mice (age: 6–8 weeks)	20, 50 mg/kg in vivo 0, 10, 20, 40 µM in vitro	[276]
	Anti-inflammatory effects	C57BL/6 mice	0.4, 0.8 mM	[277]
Licochalcone B	Anti-tumor effects	HepG2 cell	120 μM	[278]
	melanoma	B16F0 cell	5, 7.5, 10, 12.5, 15 mg/L	[279]
Quercetin-3-O-β-D-glucoside	Anti-Oxidative	-	-	[280]
Genistein	Anti-Oxidative	Keratinocytes, fibroblasts	10, 1, 100 μM	[281]
	Osteoarthritis	Human chondrocytes	0, 5, 10, 50, 100 μM/mL	[282]
	Heart protective effect	H9c2 cell male SD rat (weight: 180–200 g)	5 μM in vitro 20, 40mg/kg in vivo	[283]
	injury of the kidney	male SD rat (weight: 180–210 g)	30 mg/kg	[284]
Glycitin	Antiallergic	osteoclasts cell	10 nM	[285]
	Anti-tumor effects	U87MG cell	50 μM	[286]
Tiliroside	Anti-tumor effects	BT-549, MDA-MB-46, SK-BR-3, MCF-7, MCF-10A cell	100, 150 μM	[286]
	Anti-inflammatory effects	macrophages female C57BL/6 mice, male BALB/c mice (age: 6–8 weeks)	10, 20, 40 μM in vitro 25, 50 mg/kg in vivo	[287]
	Anti-Oxidative	female Wistar rat (weight: 180–200 g) Swiss female mice (weight: 25–30 g)	50 mg/kg	[288]
Gallic acid	Neuroprotection	male Wistar rat (weight: 250–300 g)	100 mg/kg	[289]
	Anti-Oxidative	Male ICR mice (age: 8 weeks)	100 mg/kg	[290]
	Bone Tissue Regeneration	Female SD rat (weight: 200 g)	1, 5, 25, 100 μM	[291]
	Liver Injury	male C57BL/6J mice (age: 8–10 weeks)	5, 20 mg kg	[292]
	Anti-tumor effects	22 Rv1, DU 145, PWR-1E cell	25, 50, 75 μM	[18]
	Anti-inflammatory effects	Synovial fibroblasts	40, 60, 80 μM	[293]
Agroastragaloside V	Anti-inflammatory effects	RAW 264.7 macrophages	-	[293]
Agroastragalosides I	Anti-inflammatory effects	RAW 264.7 macrophages	-	[293]
Agroastragalosides II	Anti-inflammatory effects	RAW 264.7 macrophages	-	[293]
Astragaloside IV	Anti-inflammatory effects	male SD rat (weight: 180–200 g)	40, 80 mg/kg	[294]
	Anti-tumor effects	RAW264.7 cell	800, 400, 200, 100, 50, 25, 0 μg/mL	[295]
	Immunomodulating effects	PAM cell	200, 100, 50, 25, 12.5, 6.25 μg/mL	[179]
	Anti-tumor effects	RWPE-1, PC3 cell male BALB/c nude mice (age: 4–6 weeks)	20 μmol/L in vitro, 20 μg/mL in vivo	[296]
	ameliorate atherosclerosis	male ApoE-/- mice, male C57BL/6J mice (weight: 20 ± 2 g)	5 mg/kg	[297]
	attenuates renal injury	HK-2 cell, male SD rat (weight: 170 ± 10g)	20, 40, 80 μM 20, 40, 80 mg/kg in vivo	[298]
	Improving cardiac function	male C57BL/6J mice (weight: 22 ± 2 g, age: 4–6 weeks)	40 mg/kg	[299]
	against myocardial fibrosis	male C57BL/6J mice (weight: 20–22 g, age: 7–8 weeks)	100, 200mg/kg	[300]
Astragaloside II	hepatoma	Hep G2 cell	20, 40, 80 μmol/L	[301]
	protect renal	male SD rat (age: 8 weeks)	3.2, 6.4 mg/kg	[302]
Cycloastragenol	Anti-inflammatory	BMDM cell female C57BL/6 mice (age: 6–8 weeks)	3, 10, 30 μM in vitro 12.5, 25, 50 mg/kg in vivo	[303]
	Preventing osteoporosis	MC3T3-E1 cell, male SD rat (age: 9 weeks)	0.03, 0.1, 0.3 μM	[304]
	Neuroprotection	male C57BL/6 mice (weight: 23–26 g)	5, 10, 20 mg/kg	[305]
	Anti-myocardial fibrosis	Cardiac fibroblasts, male BALB/c mice (weight: 24–25 g, age: 10 weeks)	0, 15.625, 25, 31.25, 50, 62.5 100 μg/mL in vitro 31.25, 62.5, 100, 200 mg/kg in vivo	[306]
	gastric cancer	SNU-1, SNU-16 cell	0, 1, 5, 10, 30, 50 μM	[307]
	Anti-aging	famale Kunming mice (weight: 22 ± 2 g)	2.5, 5.0, 10 mg/kg	[308]
Isoastragaloside II	Anti-inflammatory	-	-	[117]
Soyasapogenol B	Liver protection	Male BALB/c mice -	80mg/kg -	[309]
	Memory Impairment	BV-2, SH-SY5Y Cell male ICR mice (weight: 25–28 g, age: 6 weeks)	1, 10, 20 mg/kg in vivo, 5, 10 μM in vitro	[310]
Soyasaponin I	Anti-tumor effects	MCF-7, MDA-MB-231 cell	50, 70 μM	[311]
	Anti-inflammatory	IPEC-J2 cell female BALB/c mice	10 μM in vitro, 20 mg/kg in vivo	[312]

Note: concetration.

## 7. Computer-Aided Drug Design Research

**Figure 8:**
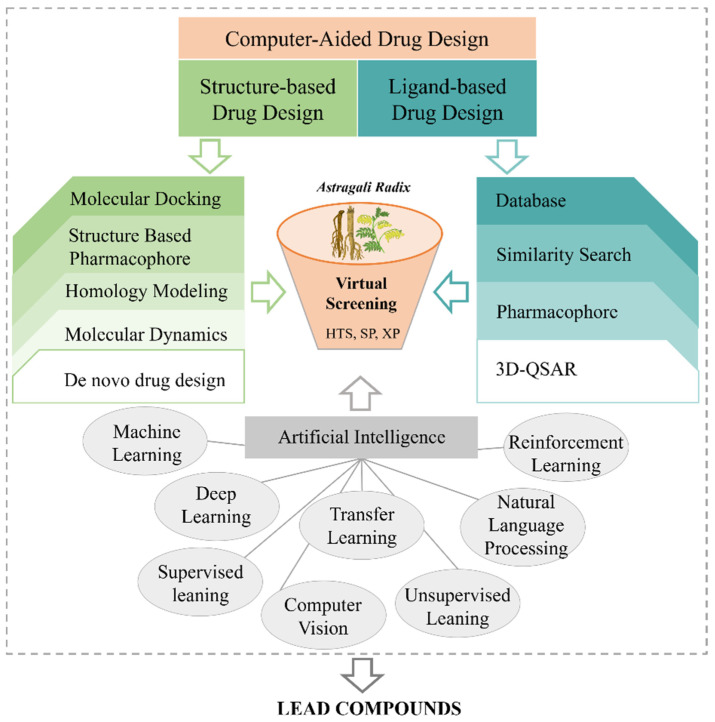
Computer-aided drug design aided the durg development of AR.

As a renowned practice of natural medicine, TCM employs a personalized and holistic approach to treat diseases through the use of natural medical products. It offers an extensive pool of potential therapeutic candidates, leveraging the vast chemical structure space of its components. However, despite the significant potential of TCM in drug discovery, traditional methods of identifying new drugs from this rich resource have proven to be challenging [313,314]. Recently, advancements in network pharmacology, complex networks, computer-aided drug design (CADD), and other methodologies have revolutionized TCM research, particularly in the discovery and optimization of lead compounds (Figure 8) [315]. These methods not only overcome the limitations of animal pharmacological experiments but also significantly enhance the efficiency and success rate of scientific research [316].

### 7.1. Discovery of Lead Compounds

Computer-aided drug design and artificial intelligence technology play key roles in the discovery of lead compounds, accelerating the development of new drugs through efficient screening and optimization [11]. The chemical constituents of AR were collected, and reverse docking, target prediction, network pharmacological analysis, molecular docking, and virtual screening were performed to identify the potential active ingredients as lead compounds for further evaluation and optimization.

Network pharmacology explores disease development from a systems biology perspective, elucidates drug-body interactions from a holistic viewpoint, and guides the discovery of new drugs [317]. Recent studies have utilized databases to collect the chemical constituents of AR, constructing networks to predict its material basis and molecular mechanisms for treating colon cancer [318]. Molecular docking, a crucial virtual screening method, predicts the interactions between TCM molecules and target proteins, allowing for the identification of potential lead compounds and facilitating the efficient screening of small molecules in TCM [319]. In 2024, Chen et al. explored the active components and molecular mechanisms of AR in heart failure by integrating component-signal-target networks with molecular docking [320].

In recent years, molecular dynamics simulations have also been incorporated into molecular screening to enhance accuracy [321]. Additionally, Artificial intelligence (AI) methods, such as graph neural networks, have also been applied in TCM research [322]. For instance, Zhang et al. identified the authenticity of more than 160 AR samples using a random-weight neural network, further demonstrating that artificial intelligence algorithms, including graph neural networks, hold significant potential for modernizing TCM [322]. Additionally, by combining machine learning algorithms with molecular simulation techniques, researchers screened the Specs database to identify potential αvβ3 integrin inhibitors. Systematic structural modifications and in vitro validation revealed that compound C19-9 exhibits significant anti-tumor activity [323]. These findings underscore the transformative potential of AI-driven methodologies, particularly machine learning-based approaches, for modern drug discovery and pharmaceutical innovation.

### 7.2. Discovery Potential Drug Targets of AR

A comprehensive understanding of the active component structures of AR facilitated constituent profiling, reverse docking searches, and Pharmacophore Construction to identify potential pharmacodynamic targets. Reverse docking computationally simulates the binding of small molecules to multiple proteins and predicts their most likely biological targets [324]. This technique has been extensively used to discover new targets for existing drugs and natural constituents [325]. For example, Kichul Park et al. applied reverse docking to ginsenosides, the active ingredients in Korean ginseng, identifying four potential targets and evaluating their potential toxicity and side effects [326]. Thus, reverse docking offers a novel approach for advancing drug research in TCM.

Pharmacophore construction represents the atomic or molecular features of a drug that facilitate non-bond interactions, such as hydrogen bonding, electrostatic interactions, and hydrophobicity with receptor-binding sites, along with their spatial arrangements [327]. Pharmacophore models have been employed to identify targets through reverse screening in target interaction databases [328]. For instance, Tang et al. matched AS-IV with multiple pharmacophore models representing various target proteins to determine their potential targets [329]. These computational approaches not only enhance the efficiency of drug discovery but also provide deeper insights into the molecular mechanisms of compounds from TCM. Future research should focus on refining these computational methods and integrating them with experimental validation to accelerate the development of novel therapeutics and advance the modernization of AR.

## 8. Conclusions and Future Direction Discussion

As research on Astragali Radix (AR) progresses, its active constituents have long been recognized as pleiotropic agents for treating various acute and chronic diseases. The diverse pharmacological properties of AR components make it a promising therapeutic option for multiple conditions [330]. Modern pharmacological studies have demonstrated that AR exhibits anti-tumor, antioxidant, cardiovascular-protective, immunomodulatory, anti-inflammatory, anti-diabetic, and neuroprotective effects, among others [3,4,5,6,7,8,9]. This review offers a comprehensive analysis of AR, covering its traditional use, botanical characteristics, drug formulation, chemical composition, and modern pharmacological properties. This review serves as a valuable resource for researchers seeking a deeper understanding of AR and its pharmaceutical potential.

AR, a renowned traditional Chinese medicinal herb, has been extensively utilized in the treatment of a wide array of diseases. Despite significant advancements in AR research, some problems remain. AR is composed of a complex array of chemical constituents, with the content of bioactive constituents varying among different varieties of AR. The extraction and processing methods affect the effective constituents of AR. In contrast, after entering the human body, the constituents of TCM are metabolized and distributed, ultimately entering the bloodstream. The identification of the components in the blood is crucial for the efficacy and safety of AR. The identification and quantification of these blood-absorbed constituents are critical for evaluating the pharmacological effects and safety profiles of AR. Therefore, it is important to control the quality of AR and determine its active ingredients. Although the pharmacological effects of AR have been extensively studied, based on the theory of TCM, we need to focus on the main effects of AR. Moreover, TCM has the characteristics of multi-component, multi-target, and multi-pathway, and AR also affects multiple pathways through multiple components and targets. Based on the primary TCM efficacy of AR, such as tonifying qi, elevating yang, and consolidating the body’s surface to reduce sweating, research should focus on its roles in immune regulation, metabolic function, and other related areas. By combining TCM theories with modern scientific approaches, studies can ex-plore the mechanisms of AR in enhancing immune responses, improving metabolic homeostasis, and addressing related disorders, thereby advancing its application in integrative medicine and contributing to the modernization of AR.

Furthermore, the field of TCM has entered a new era of development. CADD and AI have been increasingly integrated into modern TCM research. AR contains complex chemical constituents; it is necessary to build an intelligent database to summarize the constituents of AR. Conversely, there is a lack of in-depth calculation and research on the absolute configuration and medicinal properties of sugar-containing substances in AR. Molecular docking, 3D-QSAR, and similarity search have been widely used to identify effective molecules and potential targets of AR. Advanced techniques such as molecular docking, three-dimensional quantitative structure-activity relationship (3D-QSAR) modeling, and similarity searching have been widely employed to identify bioactive molecules in Astragalus and their potential therapeutic targets. Computational simulation technologies have emerged as powerful tools for elucidating the dynamic interaction mechanisms between AR’s bioactive constituents and their pharmacological targets. These technologies not only predict the binding sites and affinities between active constituents and targets but also simulate the dynamic behavior of drug molecules within biological environments, providing profound insights into their mechanisms of action and facilitating the development of targeted therapies. These advanced technologies provide a pathway for the modernization of AR. By leveraging these methods, researchers will be able to delve deeper into the mechanisms of action of AR in treating diseases, providing a more comprehensive understanding of the intricate molecular interactions between AR constituents and their targets. These efforts will provide valuable insights into the pharmacological mechanisms of AR.

In addition, clinical studies have demonstrated that APS injections effectively enhance the immune response. When used in combination with other chemical drugs, APS has been shown to amplify anti-tumor effects while reducing adverse reactions [331]. Similarly, AS-IV injections have exhibited significant efficacy in treating cardiovascular diseases. However, due to side effects and poor oral bioavailability, its development was discontinued during preclinical trials [332]. These findings suggest that the active compounds derived from Astragalus hold substantial potential for further exploration. Moreover, the integration of advanced methodologies, such as complex network analysis and CADD, has significantly improved the efficiency and success rate of drug development in AR-related research. These cutting-edge approaches offer promising prospects for screening active compounds and elucidating the mechanisms of action of Astragalus. Ongoing advancements in modern science and technology continue to facilitate the exploration and global dissemination of AR-based therapies.

## Figures and Tables

**Table 6:** Others isolated from AM and AMM.

No.	Name	Structure	Species	References
**285**	5-hydroxymethyl-2-furancarboxylic acid	1	AMM	[66]
**286**	1-(1H-pyrrol-2-yl)-ethanoe	2	AMM	[66]
**287**	5-methoxy-furan-2-carbaldehyde	3	AMM	[66]
**288**	furan-2-carbonic acid	4	AMM	[66]
**289**	4-hydroxy-benzoic acid	5	AMM	[66]
**290**	vanillic acid	6	AMM	[66]
**291**	uridine	7	AMM	[66]
**292**	adenosine	8	AMM	[78]
**293**	azelaic acid	9	AMM	[66]
**294**	hexa-2,4-dienedioic acid	10	AMM	[66]
**295**	d-3-O-methyl-chiro-inositol	11	AMM	[66]
**296**	adenine	12	AMM	[120]
**297**	guanosine	13	AMM	[120]
**298**	gluceryl α-mono-stearate	14	AMM	[79]
**299**	glucose	15	AMM	[88]
**300**	sucrose	16	AMM	[78]
**301**	monopalmitin	17	AMM	[78]
**302**	emodin	18	AMM	[86]
**303**	2,6-dimethoxy-4-hydroxyphenyl-1-O-β-D–glucopyranoside	19	AM	[69]
**304**	gentisin	20	AM	[78]
**305**	caffeic acid	21	AM	[82]
**306**	ferulic acid	22	AM	[82]
**307**	chlorogenic acid	23	AM	[82]
**308**	4-Hydroxybenzoic acid	24	AM	[82]
**309**	betaine	25	AM	[82]
**310**	vitamin B2	26	AM	[82]
**311**	niacin	27	AM	[82]
**312**	uracil	28	AM	[82]
**313**	coumarin	29	AM	[82]
**314**	6,7-Dihydroxycoumarin (Esculetin)	30	AM	[82]
**315**	6-Methylcoumarin	31	AM	[82]
**316**	13-Hydroxy-9,11-octadecadienoic acid	32	AM	[82]
**317**	Linoleic acid	33	AM	[82]
**318**	Palmitic acid	34	AM	[82]
**319**	Daucosterol	35	AM	[82]
**340**	Syringaresinol	36	AM	[82]
**341**	8,9-trans-8-hydroxymethyl-3′4′-dihydro-5′-carbaldehyde-1H-pyrrodo [2′1′-c]-1,7-dioxa-4-aza-spiro-[5,4]-9-decanol	37	AM	[66]
**342**	3′, 4′-Dihydro-5′-carbaldehyde-1H-pyrodo[2′,1′-c]-1,7-dioxa-4-aza-spiro-[5,4]-9-decanol	38	AM	[66]
**343**	2-3′,4′-Dihydroxy-(Z)-1′-butene-5-2-3′-4′-trihydroxy-butane-pyrazine	39	AM	[66]
**344**	gallic acid	40	AM	[84]
**345**	1-(2-Oxo-tetrahydro-furan-3-yl-)-5-[(2-oxo-tetrahydro-furan-3-3ylamino)-methli]-1H-pyrrole-2-carbaldehyde	41	AM	[66]
**346**	Adenosine	42	AM	[66]

## Data Availability

No new data were created or analyzed in this study. Data sharing is not applicable.

## Abbreviations

The following abbreviations are used in this manuscript:

AR	Astragali Radix
AMM	*Astragalus membranaceus* (Fisch.) Bge. var. *mongholicus* (Bge.) Hsiao
AM	*Astragalus membranaceus* (Fisch.) Bge
APS	Astragalus Polysaccharide
AS-IV	Astragaloside IV
CAT	Catalase
CD	cluster of differentiation
CADD	Computer-aided drug design
DCs	Dendritic cells
GP	glycogen phosphorylase
GSH	glutathione
GSH-Px	Glutathione Peroxidase
G-6-Pase	glucose-6-phosphatase
IL-6	interleukin-6
IL-1β	interleukin-1β
IL-8	interleukin-8
IFN-γ	interferon-γ
IR	insulin receptor
IRS	insulin receptor substrate
LPS	lipopolysaccharide
MPO	Myeloperoxidase
MDA	malondialdehyde
NF-κB	nuclear factor kappa-B
NO	nitric oxide
ORAC	absorbance capacity
PTP1B	Protein Tyrosine Phosphatase 1B
SIRT1	silent information regulator 1
SOD	superoxide dismutase
TAMs	tumor-associated Macrophages
TNF-α	tumor necrosis factor-α
TCM	Traditional Chinese medicine
